# Modulation-doping a correlated electron insulator

**DOI:** 10.1038/s41467-023-41816-3

**Published:** 2023-10-05

**Authors:** Debasish Mondal, Smruti Rekha Mahapatra, Abigail M. Derrico, Rajeev Kumar Rai, Jay R. Paudel, Christoph Schlueter, Andrei Gloskovskii, Rajdeep Banerjee, Atsushi Hariki, Frank M. F. DeGroot, D. D. Sarma, Awadhesh Narayan, Pavan Nukala, Alexander X. Gray, Naga Phani B. Aetukuri

**Affiliations:** 1 Solid State and Structural Chemistry Unit, Indian Institute of Science, Bengaluru, Karnataka India; 2 Department of Physics, Temple University, Philadelphia, PA USA; 3 Centre for Nano Science and Engineering, Indian Institute of Science, Bangalore, Karnataka India; 4 Deutsches Elektronen-Synchrotron, DESY Hamburg, Germany; 5 Department of Physics and Electronics, Graduate School of Engineering, Osaka Metropolitan University, Osaka, Japan; 6 Utrecht University, Inorganic Chemistry and Catalysis Group Universiteitsweg 99, Utrecht, The Netherlands

**Keywords:** Electronic properties and materials, Phase transitions and critical phenomena

## Abstract

Correlated electron materials (CEMs) host a rich variety of condensed matter phases. Vanadium dioxide (VO_2_) is a prototypical CEM with a temperature-dependent metal-to-insulator (MIT) transition with a concomitant crystal symmetry change. External control of MIT in VO_2_—especially without inducing structural changes—has been a long-standing challenge. In this work, we design and synthesize modulation-doped VO_2_-based thin film heterostructures that closely emulate a textbook example of filling control in a correlated electron insulator. Using a combination of charge transport, hard X-ray photoelectron spectroscopy, and structural characterization, we show that the insulating state can be doped to achieve carrier densities greater than 5 × 10^21^ cm^−3^ without inducing any measurable structural changes. We find that the MIT temperature (T_MIT_) continuously decreases with increasing carrier concentration. Remarkably, the insulating state is robust even at doping concentrations as high as ~0.2 e^−^/vanadium. Finally, our work reveals modulation-doping as a viable method for electronic control of phase transitions in correlated electron oxides with the potential for use in future devices based on electric-field controlled phase transitions.

## Abstract (web summary)

The metal-insulator transition in VO2 is concomitant with the structural transition, making purely electrical control challenging. Here the authors use a modulation-doped heterostructure to demonstrate modulation of the transition temperature with doping, without introducing structural changes.

## Introduction

Strong electron-electron correlations within narrow d- or f-orbitals underpin a variety of condensed matter phenomena, such as metal-to-insulator transitions (MITs), high-temperature superconductivity, magnetism, and multiferroicity, often observed in correlated electron materials (CEMs)^1,2^. VO_2_ is a prototypical example of a CEM with a temperature-dependent metal-to-insulator phase transition. The electronic phase transition in bulk VO_2_, which occurs at an MIT temperature (T_MIT_) of ~340 K, is accompanied by a structural phase transition from a metallic rutile phase to an insulating monoclinic phase^3,4^.

The origin of the MIT in VO_2_—whether gap-opening is driven by the symmetry-lowering structural transition or by electron-electron correlations—has been widely studied^5–8^. In particular, there is widespread interest in the nature of the insulating state and its external control via doping^9,10^, strain^11^, oxygen vacancy creation^12^, hydrogenation^13^, light-and-pulse-induced modulation^14,15^, and via electric-fields in a field-effect transistor geometry^16–18^.

For example, n-type doping of VO_2_ with dopants such as W^6+^, Mo^5+^, and Nb^5+^ was shown to decrease T_MIT_ and stabilize the metallic phase^9,10,19^. By contrast, p-type doping of VO_2_ with dopants such as Cr^3+^, Ga^3+^_,_ and Al^3+^ was shown to increase T_MIT_, thereby stabilizing the insulating phase^20–22^. Similarly, both oxygen vacancy creation and hydrogenation were shown to n-dope VO_2_ and decrease T_MIT_^12,13,23^. Finally, in VO_2_ thin films, macroscopic tensile strain along the rutile a-axis was also shown to decrease T_MIT_^11,12^.

In all these previous approaches, modulation of T_MIT_ was always associated with macroscopic changes to the lattice parameters (due to strain) and/or dopant-induced local structural distortions^9,10,12,13,20,23^. In such experiments, where both the lattice strain and carrier concentration change, it is challenging and, sometimes, impossible to disentangle the role of carrier concentration changes from the role of lattice strain. For instance, in the case of W-doped VO_2_, an increase in W-doping concentration increases both the carrier density and lattice strain^24^.

Other techniques utilizing external stimuli, such as electric-field-induced metallization of VO_2_ in a field-effect transistor geometry, could, in theory, enable the modulation of its conductivity without inducing macroscopic structural changes. However, previous attempts at electric-field-driven metallization of VO_2_ were not successful^12,16,17,25,26^. For example, attempts at modulating the MIT in VO_2_ in a field effect transistor geometry yielded less than a 1 K change in T_MIT_^27,28^. The weak response of T_MIT_ of VO_2_ to external electric field, even when gated through high-K dielectrics, was attributed to the presence of strong correlations in the insulating VO_2_ phase^16^. Further, ionic-liquid gating of VO_2_, which could enable accessibility to large interfacial electric-fields, led to oxygen vacancy creation and/or hydrogenation of VO_2_^12,16^. Heterostructures with differing compositions such as for example VO_2_/W_x_V_1-x_O_2_ based heterostructure thin films showed a larger change in T_MIT_^29^. However, these changes are related to elemental doping driven by a chemical potential mismatch of the dopant-ion (W^6+^ in this specific case). By stark contrast, we propose modulation-doping of VO_2_ using electronic chemical potential differences at oxide heterostructures.

Modulation- or remote-doping of oxide semiconductors is an alternative method for achieving high dopant carrier densities without inducing local structural distortions^30–33^. In modulation-doping, a chemical potential mismatch between a high band gap heavily doped layer (dopant-layer) and a lower band gap undoped layer (channel) leads to charge transfer from the heavily doped dopant-layer to the undoped channel. In general, the dopant layer and the channel are spatially separated by a barrier (or a spacer) layer that kinetically limits the interdiffusion of the dopants from the dopant layer to the channel layer while allowing charge transfer via quantum mechanical tunneling^34,35^.

Modulation-doping was successfully applied to semiconductors and band-insulating oxides such as ZnO and SrTiO_3_^31,33,36–39^. However, modulation-doping of correlated electron insulators has had limited success. For example, Stemmer and colleagues reported modulation-doping of NdNiO_3_, but this did not lead to any significant changes in T_MIT_ of the nickelate^31^. Whether modulation-doping could be a generic approach to induce phase transitions in oxides is unclear and several key questions remain unanswered. For example, how do bands evolve in correlated oxides as a function of doping? Can a rigid band model be applied to understand doping in oxides? How sensitive are the ground state properties in correlated oxides to carrier doping?

In this work, we address some of these open questions using the MIT in VO_2_ as a model system. We propose a modulation-doped heterostructure to n-dope VO_2_ without inducing any structural distortions. Commonly in modulation-doping, an epitaxial structure is grown with a spacer, and the dopant layers are epitaxially matched to the semiconducting channel layer. Note that both the spacer and dopant layers must be insulating with a bandgap that is higher than that of the channel layer. However, the only stable rutile oxide that is insulating with a compatible band mismatch that allows modulation-doping of VO_2_ is TiO_2_. The other rutile oxides such as CrO_2_, RuO_2_, and IrO_2_ are metallic and therefore not compatible as dopant layers^40,41^.

As an additional challenge, oxygen lattice continuity in epitaxial structures might also lead to oxygen vacancy diffusion across the layers^23,42,43^. We note that oxygen vacancy formation, which was shown to affect the MIT in VO_2_, is commonly observed in transition metal oxides^12,44,45^. Thus, in order to prevent oxygen vacancy diffusion across the wide band-gap spacer layer as well as to circumvent the lack of lattice-matching insulating rutile oxides, we have gone away from epitaxially-matched modulation-doped heterostructures. Instead, we propose an amorphous LaAlO_3_ (LAO) layer (with a reported electronic band gap of ~5.6 eV)^46^ as the spacer layer. Since LAO has a low oxygen vacancy-diffusivity^47^, we use an amorphous oxygen-deficient TiO_2-x_ as the dopant layer. Stoichiometric TiO_2_ has a bandgap of ~3 eV^48^ and TiO_2-x_ is n-type conducting. Using TiO_2-x_ instead of a conventionally doped TiO_2_ such as Nb-doped TiO_2_, significantly simplified heterostructure deposition. Furthermore, this approach avoids the interdiffusion of metallic dopants such as Nb in Nb-doped TiO_2_ and the associated unintentional doping of VO_2_.

**Fig. 1:**
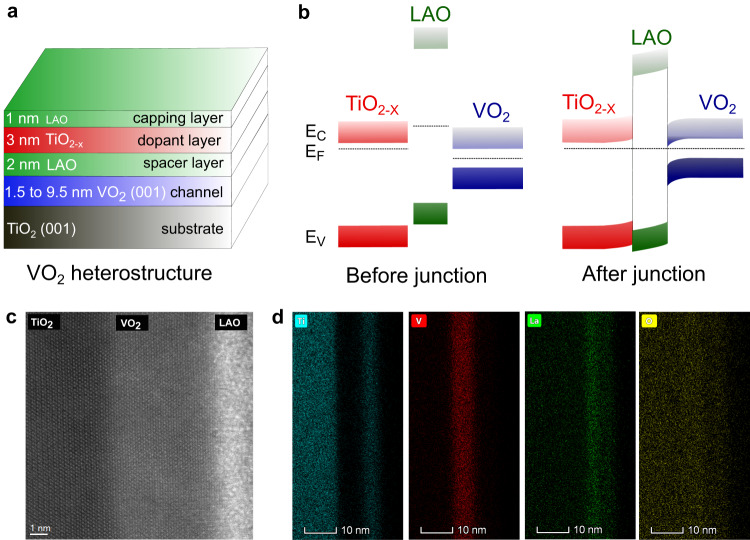
Structure of Modulation-doped VO_2_ heterostructures. **a** Schematic diagram of the heterostructures used in this work. The thickness of VO_2_ is varied while the thicknesses of all the other layers are as mentioned in the schematic. **b** Schematic energy band diagram for a VO_2_/LAO/TiO_2-x_ heterostructure before and after the junction formation. Electron accumulation is expected based on the known band offsets. The color intensities are chosen to be proportional to expected electron densities for better visualization. E_C_, E_V_, and E_F_ indicate the conduction band edge, valence band edge, and Fermi level, respectively. **c** High-resolution cross-sectional high-angle annular dark-field scanning transmission electron microscopy (HAADF-STEM) image showing abrupt interfaces between TiO_2_ substrate and VO_2_ film and VO_2_ film and the amorphous LAO spacer layer. **d** Elemental mapping using energy dispersive x-ray spectroscopy (EDS) showing the various layers in the heterostructure. Note that the scales of **c** and **d** are different.

The modulation-doped structure for all samples used in this work is comprised of a VO_2_ channel layer, a 2 nm thick LAO spacer layer, and a 3 nm thick TiO_2-x_ dopant layer, as shown schematically in Fig. 1a. All heterostructures were capped with a 1 nm thick LAO layer to prevent dopant passivation from atmospheric impurities as well as oxidation of the TiO_2-x_ dopant layer. Fermi level alignment across the structure is expected to lead to an electron accumulation region at the LAO/VO_2_ interface. Expected band-alignments for this type-I heterojunction before and after heterostructure formation are shown in Fig. 1b.

## Results

To experimentally realize the proposed modulation-doped structure, all samples were grown using pulsed laser deposition (PLD) on single-crystalline TiO_2_ (001) substrates. VO_2_ was deposited at 425 °C, while all the other amorphous layers were deposited at room temperature (see methods section for details). It is important to note that room-temperature deposition of the spacer, dopant and capping layers also minimizes any interfacial interdiffusion. A cross-sectional scanning transmission electron microscopy (STEM) image (Fig. 1c) and the associated energy dispersive spectroscopy (EDS) elemental maps (Fig. 1d) show abrupt high-quality interfaces between the TiO_2_ substrate and the VO_2_ film, and between the film and LAO spacer. Furthermore, the STEM image of pristine VO_2_ on TiO_2_ (001) substrate shows (supplementary Fig. 1) that the epitaxial atomic arrangement of VO_2_ is identical for VO_2_ heterostructures and thin films. This data is consistent with in-situ RHEED patterns of the deposited VO_2_ films and suggests that the films are both atomically smooth and single-crystalline (Supplementary Fig. 2). The atomic force microscopy (AFM) images of the complete heterostructures further confirm the high quality of the growth by showing atomically smooth film surfaces (Supplementary Fig. 3).

To study the correlation between modulation-doping-induced carrier density changes and the changes in the MIT characteristics, we deposited several modulation-doped heterostructures with varying thicknesses of the VO_2_ layer ranging from 1.5 nm to 9.5 nm, while keeping the thickness of the TiO_2-x_ layer unchanged at 3 nm. Thomas-Fermi screening lengths in VO_2_ are expected to be on the order of 1 nm (Supplementary Note 1) and, therefore, the highest n-type carrier densities are expected for the lowest VO_2_ film thickness used in this study (~1.5 nm). Heterostructures with VO_2_ films thinner than ~1.5 nm were not attempted due to the expected titanium interdiffusion at the VO_2_ film and TiO_2_ substrate interface^49,50^. We note that interfacial titanium interdiffusion will be present in thicker films as well. However, at VO_2_ thicknesses greater than 1.5 nm, there is still an observable MIT.

**Fig. 2:**
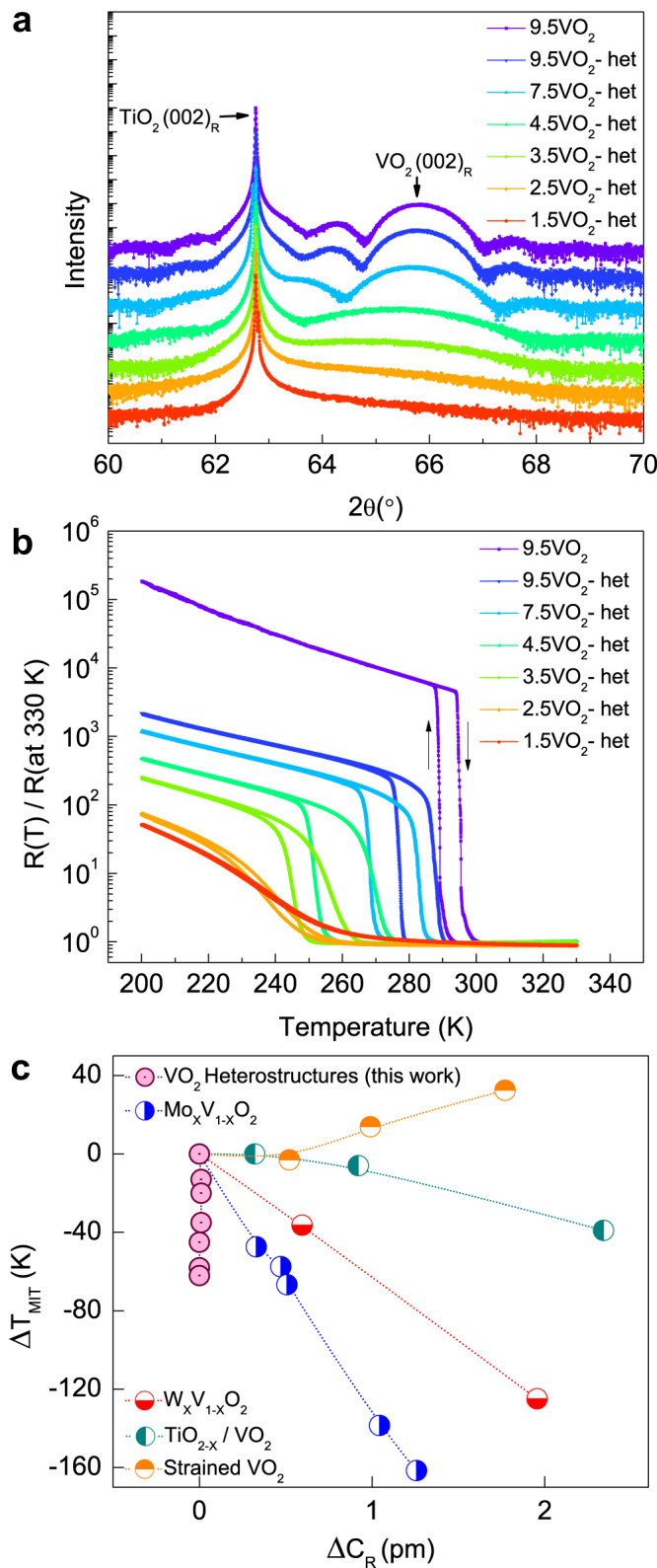
XRD and temperature-dependent electrical transport in VO_2_ heterostructures. **a** High-resolution θ-2θ XRD spectra for 9.5 nm VO_2_ thin film and for VO_2_ heterostructures with varying thicknesses. For nomenclature simplicity, we distinguish VO_2_ thin films and heterostructures with a VO_2_ thickness of ‘t’ as tVO_2_ and tVO_2_-het, respectively. **b** Resistance versus temperature plots for the same set of samples as shown in **a**. Resistance values presented here are normalized to the resistance at 330 K (also see Supplementary Fig. 7). **c** A comparison of the changes in T_MIT_ versus the changes in the rutile C-axis lattice parameter (ΔT_MIT_ vs ΔC_R_) for this work and other previously published work using W- and Mo-doping^9,10^, oxygen vacancy doping^22^ and strain^11^. The relative changes are compared to the undoped and unstrained states in the case of bulk doping and for strained VO_2_, respectively. [also see Supplementary Table 1].

A summary of the θ–2θ X-ray diffraction (XRD) measurements, performed at room temperature, for a 9.5 nm VO_2_ film and the VO_2_/LAO/TiO_2-x_/LAO heterostructures on TiO_2_(001) substrates are shown in Fig. 2a. Clearly, the angular positions of the VO_2_(002) Bragg reflection peaks are identical for both the 9.5 nm VO_2_ film (purple spectrum) and the 9.5 nm VO_2_ heterostructure (blue spectrum). Furthermore, it is evident that the angular position of the Bragg reflection is essentially independent of the thickness of the VO_2_ film in the heterostructure. Additionally, there were no significant changes in the θ-2θ X-ray diffractograms between VO_2_ films and heterostructures with the same VO_2_ thickness (Supplementary Fig. 4). Reciprocal space maps also confirm that all samples are coherently strained in the plane of the TiO_2_(001) substrate. The out-of-plane rutile c-axis lattice parameter is identical for the thin film and the heterostructures for all thicknesses of VO_2_ (Supplementary Fig. 5). Based on θ-2θ XRD measurements, reciprocal space maps, and cross-sectional STEM imaging we conclude that there are no changes in the lattice parameters between VO_2_ films and heterostructures. And therefore, changes in strain cannot account for the reduction in T_MIT_ observed in modulation-doped VO_2_ heterostructures (see Supplementary Figs. 1 and 4 and Supplementary Table 1). We also note that the reflections for the LAO spacer and capping layers and the TiO_2-x_ dopant layer are absent, suggesting that these layers are not crystalline.

Next, we discuss the variations in the MIT characteristics for the same set of samples as used in the XRD studies. As shown in Fig. 2b, T_MIT_ systematically decreases with decreasing VO_2_ thickness in the heterostructure. Note that the decrease in T_MIT_ for thin films of VO_2_ is thickness-independent (Supplementary Fig. 6), suggesting that the decrease in T_MIT_ for VO_2_ heterostructures is intrinsic to heterostructure formation. Furthermore, the sheet resistance of VO_2_ heterostructures in the insulating state also decreases (Supplementary Fig. 7). Except for the heterostructure with the thinnest VO_2_ layer (1.5 nm), all films continue to show a positive temperature coefficient of resistance, suggesting metallicity above T_MIT_.

We summarize our observations and compare the changes in T_MIT_ with other previously published studies in Fig. 2c (also see Supplementary Fig. 8 for details regarding T_MIT_ calculation). Significantly, there is a nearly 60 K change in T_MIT_ for the thinnest heterostructures without any measurable changes to the rutile C-axis. In contrast, any comparable change in T_MIT_ in the literature is associated with ΔC_R_ greater than 0.5 pm. This demonstrates control over the MIT in VO_2_ without any measurable structural changes in the VO_2_ heterostructures proposed and synthesized in this work. We note that a decrease in T_MIT_ was observed for elemental doping of VO_2_ with n-type dopants such as W and Mo, while an increase in T_MIT_ was observed for hole-doping with elements such as Cr and Al. There is no W or Mo in any of the heterostructures in this work, and both La and/or Al doping can be ruled out because they would (if anything) lead to hole-doping resulting in an increase in the T_MIT_. This is contrary to the decrease in T_MIT_ observed in these VO_2_ heterostructures.

**Fig. 3:**
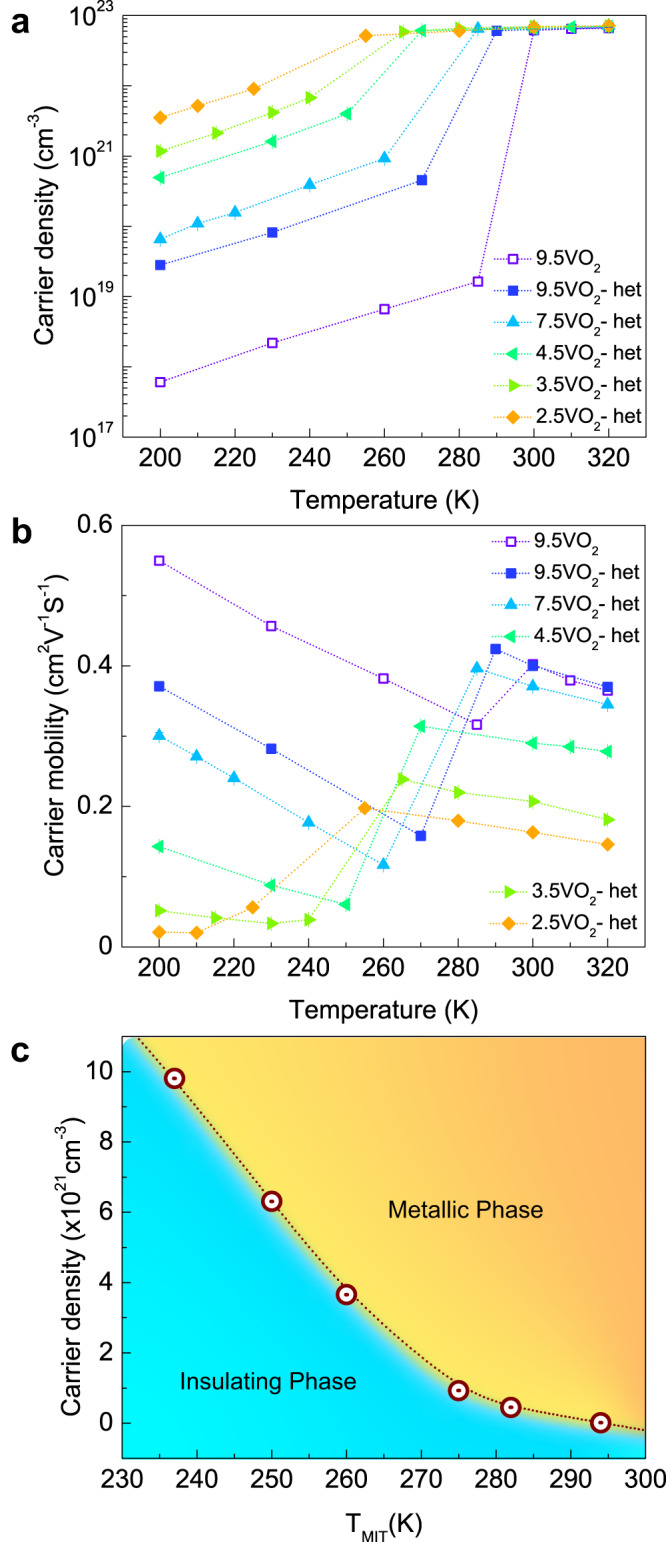
Carrier concentration-dependent MIT in VO_2_ heterostructures. Plots of temperature-dependent **a** carrier densities and **b** carrier mobilities for tVO_2_ and tVO_2_-het samples as mentioned in the legends. Carrier density in the insulating state increases with decreasing VO_2_ thickness while carrier mobility decreases. **c** A phase diagram from the results in **a**. The dotted lines connected across the data points are a guide to the eye.

To measure the extent and type of doping, we performed temperature-dependent Hall measurements. Hall measurements show an enhancement in the carrier densities in the insulating state with the Hall coefficient indicative of electron-doping (Fig. 3a). On the other hand, carrier densities in the insulating phase increased from ~6 × 10^17 ^cm^−3^ (for 9.5 nm VO_2_ thin film) to ~2.8 × 10^19 ^cm^−3^ (for 9.5 nm VO_2_ heterostructure) to a highest of ~5 × 10^21 ^ cm^−3^ for the 2.5 nm VO_2_ heterostructure. Contrastingly, the metallic state carrier densities remain identical (~6 × 10^22^ cm^−3^) across all the VO_2_ heterostructures and are consistent with previous reports^12,25^.

However, carrier mobility decreases in both the insulating and metallic states as the thickness of the VO_2_ layer decreases (Fig. 3b). In the metallic state, this is potentially due to contributions from interfacial scattering, which increases with decreasing film thickness. In the insulating state, the decrease in carrier mobility could result in part from increased electron-electron scattering and interfacial scattering. A summary of the changes in carrier concentration is plotted against T_MIT_ in Fig. 3c. There is a clear correlation between the T_MIT_ and the carrier density with the highest carrier density of ~5 × 10^21^ cm^−3^ stabilizing the metallic state of VO_2_ to a T_MIT_ of ~237 K (Supplementary Fig. 8). Importantly, the continuous increase in carrier density with decreasing VO_2_ thickness without any lattice parameter changes is suggestive of modulation-doping in VO_2_ heterostructures.

To further establish that most of the carriers are induced by modulation doping, we prepared two additional control samples. The first is a 7.5 nm VO_2_ thin film with a 2 nm LAO cap layer (7.5VO_2_-LAO) and the second is a 7.5 nm VO_2_ heterostructure with a 3 nm stoichiometric TiO_2_ layer (7.5VO_2_-LAO-TiO_2_). We compared the MIT characteristics of these two samples with the MIT characteristics of the 7.5 nm VO_2_ thin film (7.5VO_2_) and a 7.5 nm VO_2_ heterostructure with a TiO_2-x_ dopant layer (7.5VO_2_-het). A summary of sheet resistance versus temperature data is shown in Supplementary Fig. 9. The 7.5 nm VO_2_ heterostructure with a TiO_2-x_ dopant layer has the lowest sheet resistance and the lowest T_MIT_ with a T_MIT_ change of ~20 K. By contrast, the decrease in T_MIT_ was restricted to ~8 K after the deposition of the 2 nm LAO layer. Remarkably, there is no further decrease in T_MIT_ in the heterostructure with a stoichiometric TiO_2_ layer, suggesting that the TiO_2-x_ dopant layer is required for the observed large change in T_MIT_.

Consistent with this, the carrier density in the 7.5 nm VO_2_ heterostructure with the TiO_2-x_ dopant layer is ~7 × 10^19^ cm^−3^ compared to a carrier density of 2 × 10^19^ cm^−3^ for the 7.5 nm VO_2_ capped with 2 nm LAO (Supplementary Fig. 10). It is possible that amorphous (disordered) LAO could host ionized donors and enable modulation-doping^33^. However, we found that the amorphous LAO deposited for these experiments is insulating, suggesting that any ionized donors should be below the measurement threshold of electrical resistivity measurements. We estimate that such ionized donors in LAO, if any, should have a carrier density of ~10^19 ^cm^−3^ (assuming a carrier mobility of 0.01 cm^2^/V-s) or lower, putting an upper bound on the number of carriers contributed by the spacer layer.

**Fig. 4:**
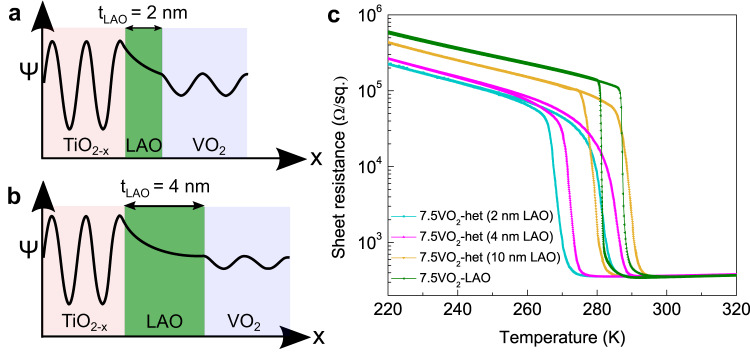
Transport characteristics of heterostructures as a function of spacer layer thickness. Schematic representation of quantum mechanical tunneling of charge carrier across the spacer layer for **a** 2 nm and **b** 4 nm thicknesses of LAO (t_LAO_). ‘Ψ’ is the electronic wave function. The schematics represent a decrease in the transfer of charge carriers from the dopant layer (TiO_2-x_ layer) to the VO_2_ layer as a function of increasing t_LAO_. **c** Resistance-Temperature plots comparing the MIT characteristics of 7.5 nm VO_2_ modulation-doped heterostructures employing t_LAO_ of 2 nm, 4 nm, and 10 nm with the MIT characteristics of a 7.5 nm VO_2_ film with a 2 nm LAO cap layer, but without any dopant layer.

As further proof of modulation-doping, we performed resistance-temperature measurements on heterostructures with varying LAO spacer layer thicknesses of 2 nm, 4 nm, and 10 nm. The thickness of VO_2_ is fixed at 7.5 nm and that of TiO_2-x_ dopant layer is fixed at 3 nm for all three heterostructures. As the thickness of the LAO layer (t_LAO_) increases, the probability of charge transfer from the TiO_2-x_ dopant layer to the VO_2_ layer decreases (Fig. 4a, b). Consistent with this, at the highest t_LAO_ of 10 nm, where the lowest amount of charge transfer is expected from the dopant layer, T_MIT_ is the closest to that of a 7.5 nm VO_2_ film with a 2 nm LAO cap layer, but without the dopant layer. Clearly, the dopant layer does not significantly affect the transition temperature when a 10 nm thick LAO spacer layer is used (Fig. 4c). By contrast for the heterostructure with t_LAO_ = 2 nm, the T_MIT_ is shifted by ~20 K as discussed earlier. Since the thicknesses of the other layers are fixed, the larger shift in T_MIT_ for the t_LAO_ = 2 nm heterostructure implies an increased charge transfer for the thinner spacer layers. This further reinforces the central conclusion that the shift in the transition temperature is enabled by modulation doping.

To probe the electronic band bending that enables electron accumulation in the VO_2_ channel layer, we performed bulk-sensitive hard X-ray photoelectron spectroscopy (HAXPES)^51^ measurements at the P22 beamline in the PETRA III synchrotron at DESY. We note that standard ultra-violet photoemission (UPS) or soft X-ray photoemission measurements cannot facilitate a probing depth sufficient to reach the VO_2_/LAO interface that is buried beneath multiple layers of the heterostructure. To capture interfacial band bending in VO_2_, HAXPES measurements were performed in both the insulating phase (at 200 K) and the metallic phase (at 310 K) for VO_2_ heterostructures with VO_2_ thicknesses of 1.5 nm, 3.5 nm, 4.5 nm, and 7.5 nm, and for a VO_2_ thin film with a thickness of 7.5 nm as a reference.

**Fig. 5:**
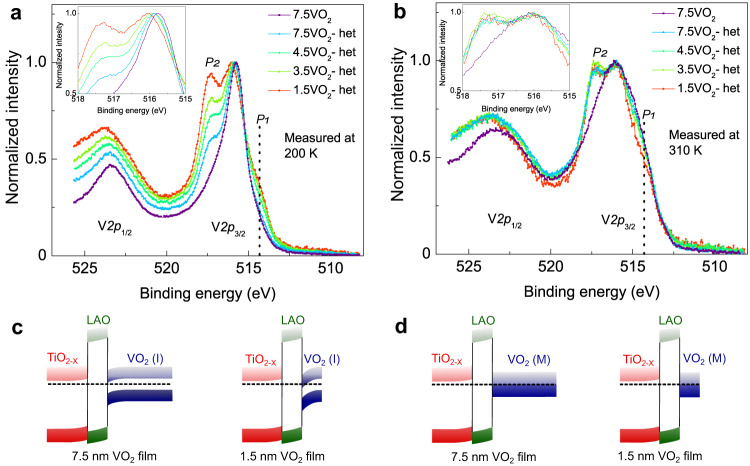
Band bending and core level spectral changes in VO_2_ heterostructures. A comparison of V *2p* core-level spectra of modulation-doped VO_2_ heterostructures for **a** the insulating (200 K) and **b** the metallic states (at 310 K). A clear shift in the V *2p* levels is seen in the insulating state spectra but not in the metallic state spectra. Schematics in **c** and **d** show the expected band-bending in the modulation-doped heterostructures for the insulating and metallic states respectively. Band-bending is expected in the insulating state and not in the metallic state. Two additional spectral features not seen in VO_2_ thin films are labeled *P1* and *P2*.

For the 7.5 nm VO_2_ film measured at 200 K (insulating state), the binding energies of the V 2*p*_3/2_ and V 2*p*_1/2_ core-level peaks were observed to be ~515.8 eV and ~523.1 eV, respectively (see Fig. 5a). These measured binding energies (see Supplementary Fig. 11 for binding energy calibration procedure) are consistent with previous reports^52–54^. Importantly, a systematic shift of the main component of the V 2*p*_3/2_ peak to higher binding energies is observed for the VO_2_ heterostructures, with the highest increase in binding energy (~250 meV) observed for the heterostructure with the thinnest VO_2_ layer (1.5 nm), as seen in the inset. This is also in agreement with the highest carrier densities and the lowest T_MIT_ being observed for the heterostructures with the thinnest VO_2_ layers. For measurements performed on VO_2_ in the metallic state, no such binding energy shift was observed (Fig. 5b and Supplementary Fig. 12). This is consistent with the complete screening of interfacial electric fields at the metallic VO_2_/LAO interface (Fig. 5c, d). The presence of binding energy shifts observed in the insulating state of VO_2_ and their absence in the metallic state of VO_2_ further support carrier doping by chemical potential shifts (modulation-doping) in the insulating state for VO_2_ heterostructures as proposed in this work.

Photoemission data also showed two remarkable features in the V 2*p* spectra. The first, labeled *P1* in Fig. 5a, b, is a lower binding energy shoulder around ~514.5 eV in the insulating and metallic states. The presence of this spectral feature at lower binding energies was proposed to signify non-local screening from coherent 3d^1^ states near E_F_ in the metallic phase of VO_2_^54,55^. Interestingly, the intensity of *P1* in the insulating state increases with increasing carrier density and decreasing VO_2_ thickness in the heterostructures. The emergence of this peak in the insulating state spectra for VO_2_ heterostructures suggests that the additional charge transferred to the VO_2_ channel layer enables non-local screening that was previously observed only in the metallic phase of VO_2_.

To further quantify the evolution of the *P1* peak across the metallic and insulating phases, we compared the metallic and insulating state spectra of 1.5 nm and 7.5 nm VO_2_ heterostructures in Supplementary Fig. 13. The intensity of the *P1* peak in the insulating state of the 7.5 nm VO_2_ heterostructure is observable but small in comparison to the *P1* peak in the metallic state (Supplementary Fig. 13a). The intensity difference spectrum shows a large difference at ~514.5 eV (Supplementary Fig. 13c) further confirming that the non-locally screened shoulder is negligibly small in the insulating state when compared to the metallic state. Remarkably, the non-locally screened shoulder is quite predominant in the insulating state spectra of 1.5 nm VO_2_ heterostructure (Supplementary Fig. 13b). The intensity difference spectra between the metallic and insulating states show a very small difference between the two phases (Supplementary Fig. 13d). These trends are consistent with the differences in the carrier densities between the metallic and insulating phases. The carrier density ratio between the metallic and insulating states is about 10 for the 1.5 nm VO_2_ heterostructure compared to about 1000 for the 7.5 nm VO_2_ heterostructure.

The second remarkable feature in the photoemission data is a high binding energy spectral feature at ~517.5 eV. This feature is associated with the V 2*p*_3/2_ peak and labeled *P2* in Fig. 5a, b. A corresponding feature is also observed for the V 2*p*_1/2_ peak but is more smeared out and appears as broadening on the higher-binding-energy side at ~525 eV. In general, higher binding energy spectral features are associated with higher oxidation states in photoemission. The presence of V^5+^ is a possibility. However, an increase in the oxidation state from V^4+^ to V^5+^ cannot explain the observed electron-doping in VO_2_ heterostructures. Generally, electron doping should decrease the V^4+^ oxidation state in VO_2,_ and therefore, an increase in the oxidation state of vanadium cannot explain the increase in electron density in the VO_2_ heterostructures. Therefore, V^5+^, even if present, has no bearing on the MIT observed in heterostructures.

*P2* was also observed in VO_2_ samples capped with 2 nm LAO (Supplementary Fig. 14). Therefore, we have also inspected the La 3*d*_5/2_ and Al 1*s* spectra to look for any chemical shifts associated with a redox or chemical reaction at the VO_2_/LAO interface. As shown in Supplementary Fig. 15, there are no observable changes to the spectra across heterostructures. Finally, interfacial oxygen vacancy creation remains a possibility. However, any oxygen vacancy creation should lead to V^3+^ and an associated low-binding-energy feature in both the metallic and insulating state spectra. However, the spectra do not show any signatures of oxygen vacancies in VO_2_ heterostructures. Therefore, we rule out any oxygen-vacancy induced carrier doping in these heterostructures.

Furthermore, the intensity of *P2* is carrier density dependent. For all V 2*p* spectra in the metallic state, where the carrier density is independent of the VO_2_ thickness, the intensity of this additional peak relative to the main V 2*p*_3/2_ peak is also independent of the VO_2_ thickness, with the intensity ratio of *P2* to V 2*p*_3/2_ being close to 1. Contrastingly, the intensity of *P2* increases with the decreasing film thickness in the insulating state of VO_2_. The intensity ratio of *P2* to V 2*p*_3/2_ approaches the intensity ratio observed for the metallic state spectra at the highest carrier density of ~5 × 10^21^ cm^−3^ in the insulating state. These carrier-density-dependent changes suggest that this new spectral feature is intrinsic to the heterostructure. However, this additional spectral feature might benefit from further spectroscopic investigation with complementary techniques such as X-ray absorption spectroscopy to confirm its origins.

Furthermore, since there is a strong correlation between electron density and *P2* peak intensity, we performed an LDA + DMFT Anderson impurity model calculation for the undoped and electron-doped VO_2_ to examine the V*2p* XPS spectral changes with electron doping. The calculation of the V*2p* XPS spectrum shows that electron doping creates a satellite peak at approximately 517.5 eV (see Supplementary Note 2 and Supplementary Fig. 16 for details of the calculations). Since the V-O covalency for the V^3+^ (d^2^) state is weaker than V^4+^ (d^1^) one, the binding energy of V^3+^ in electron-doped VO_2_ increases due to a weaker bonding and anti-bonding splitting in the XPS final states. Therefore, we assign the *P2* peak to a satellite induced by electron doping.

The combination of electron transport and HAXPES data show that VO_2_ heterostructures facilitated effective modulation-doping and carrier densities as high as 5 × 10^21^ cm^−3^ could be achieved using this approach. The highest carrier densities correspond to electron doping of ~0.2 e-/vanadium. This is an extremely high dopant density at which conventional rigid band models predict metallization in doped correlated insulators^56^.

**Fig. 6:**
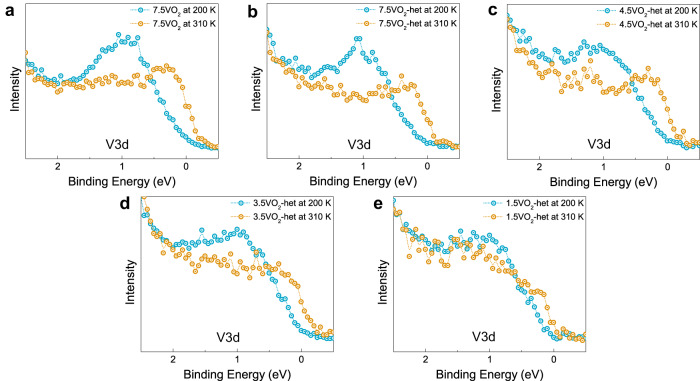
Robust MIT in VO_2_ at high carrier densities. A comparison of the V 3d valence band (VB) spectra of modulation-doped VO_2_ heterostructures for the insulating (200 K, blue) and metallic states (at 310 K, orange) for different VO_2_ film thicknesses of **a** 7.5 nm VO_2_ film and **b** 7.5 nm, **c** 4.5 nm, **d** 3.5 nm, and **e** 1.5 nm VO_2_ heterostructures. There is a clear spectral weight shift across the MIT for all the samples with the insulating state being robust even for the heterostructure with a VO_2_ thickness of 1.5 nm, corresponding to carrier doping of ~0.2 e^−^/Vanadium.

Bulk-sensitive valence-band HAXPES spectra recorded for the same set of heterostructures as discussed in Fig. 5 are shown in Fig. 6 and Supplementary Fig. 17. The insulating-state spectra for all VO_2_ heterostructures (blue) exhibit nearly zero spectral intensity at the Fermi level while an appreciable non-zero spectral intensity is observed for the higher-temperature metallic-state spectra (orange). These spectra further confirm that VO_2_ continues to undergo an MIT even in the presence of electron densities as high as ~0.2 e^−^/vanadium. The presence of MIT at such high doping levels, without any observable changes in the lattice parameters (Fig. 2a and Supplementary Fig. 4), points to a possible renormalization of the electronic structure with doping.

**Fig. 7:**
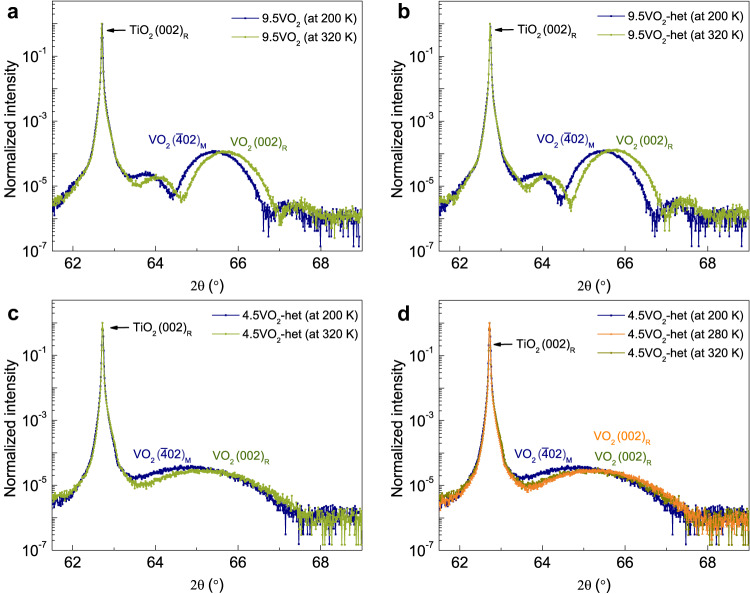
Temperature-dependent X-ray diffractograms of VO_2_ thin films and heterostructures. X-ray diffractograms of **a** 9.5VO_2_, **b** 9.5VO_2_-het, and **c** 4.5VO_2_-het measured in the insulating phase (at 200 K) and the metallic phase (at 320 K) of VO_2_. VO_2_ ($\bar{4}02$)_M_ and (002)_R_ reflections can be clearly distinguished for all the heterostructures studied in this work. The $(\bar{4}02)$_M_ peak is the out-of-plane (of the substrate) Bragg reflection in the monoclinic phase of VO_2_ while (002)_R_ is the out-of-plane reflection in the rutile phase. **d** A comparison of temperature-dependent XRD spectra of the 4.5VO_2_-het film measured at 200 K, 280 K, and 320 K. The XRD spectrum measured at 280 K shows rutile phase characteristics like the one measured at 320 K suggesting that VO_2_ remains in the rutile phase at 280 K in the 4.5 nm VO_2_ heterostructure.

What is unclear however is the presence or absence of the structural phase transition. To probe this, we performed temperature-dependent XRD measurements at 320 K (metallic phase) and 200 K (insulating phase) for a 9.5 nm VO_2_ thin film and 9.5 nm and 4.5 nm VO_2_ heterostructures. Figure 7a shows that there is a clear shift in the angular position of the out-of-plane Bragg reflection. This was attributed to the rutile to monoclinic phase transition in previous studies^11^. Similar changes to the out-of-plane Bragg reflection were also observed for the 9.5VO_2_-het and 4.5VO_2_-het across the MIT (Fig. 7b, c). Additionally, the X-ray diffractogram measured at 280 K for the 4.5VO_2_-het shows rutile phase characteristics akin to that observed for the diffractogram measured at 320 K (Fig. 7d) suggesting that the electronic phase transition is concomitant with the structural phase transition.

Moreover, in Supplementary Fig. 18, we have also compared the XRD spectra of 9.5VO_2_ and 9.5VO_2_-het both in the insulating and metallic phases. It is clear from the figure that there is an excellent overlap of the diffractograms for the film and heterostructure, including thickness fringes, for both the structural phases. This is evidence that modulation-doping decreases the phase transition temperature but does not suppress the structural transition. The presence of the structural phase transition even at carrier densities as high as 5 × 10^21^ cm^−3^ is suggestive of a strong electron-lattice coupling in VO_2_.

In summary, we demonstrated a purely electronic control of the MIT in modulation-doped VO_2_ heterostructures. Our work shows that the insulating state in VO_2_ is surprisingly robust even in the presence of electron doping as high as 0.2 e-/vanadium. Notably, all the films meet the Mott criterion (${a}_{B}\cdot {n}_{C}^{\frac{1}{3}} > 0.25$, where *a*_*B*_ is the effective Bohr radius and *n*_*C*_ is the carrier density). Therefore, metallicity is expected at all temperatures based on the carrier densities achieved in these experiments. We note that a similar robust insulating state had also been found in modulation-doped nickelate thin films^31^. Perhaps, the development of theoretical models that go beyond the conventional carrier concentration independent rigid-band models will be required to understand electronic phase transitions in correlated electron oxides. An alternate explanation could be that the insulating state is favored at lower thicknesses due to interfacial disorder-induced Anderson-like localization of carriers, which will be more pronounced for the thinnest films. Further experiments will be needed to assess whether the insulating state is stabilized by the interfacial disorder.

A remarkable feature of this work is the possibility of bulk metallization in modulation-doped VO_2_. In general, in band semiconductors such as SrTiO_3_, conductivity modulation is achieved over a thickness of 1–2 nm in the vicinity of the channel/spacer interface^33,57^. In this work, a sharp MIT is observed for heterostructures with VO_2_ thicknesses as high as 9.5 nm, which are much higher than the Thomas Fermi screening length of ~1 nm. This is suggestive of the entirety of the film being metallized at the lowered T_MIT_ after modulation-doping, even though the charge transfer densities are the highest at the interface (within 1–2 nm of the interface). While further studies are required to establish this beyond doubt, interfacial-doping induced bulk metallization of correlated electron insulators has implications for low-power electronics with high ON/OFF ratios^32,58^.

Finally, our work shows that modulation-doping is a powerful technique for achieving high carrier densities, close to those possible with elemental doping. Since our approach does not need any epitaxially matched spacer and dopant layers, it expands the library of materials that can be explored for the study of modulation-doping-induced electronic phase transitions of other related CEMs including complex oxides^2^ and pyrochlores^59^. This methodology, therefore, paves the way for exploring ‘pure’ electronic effects in correlated oxides and related systems. Such studies could also enable a fundamental understanding of band matching and relevant energy scales in complex oxides and, perhaps enable the discovery of new interfacial phases and devices that rely on phase transitions.

## Methods

Prior to deposition, single-crystalline rutile TiO_2_ (001) substrates (Shinkosa, Japan) were treated using the procedure discussed previously^60^. All thin film samples were deposited using PLD (NEOCERA) with a 248 nm KrF laser. All VO_2_ thin films (both pristine films and heterostructures) were deposited on treated TiO_2_ substrates from a sintered V_2_O_5_ target with a laser fluence of ~1.5 J/cm^2^, 8 mTorr of oxygen pressure, and a growth rate of ~4.7 × 10^−2 ^Å/pulse at a substrate temperature of 425 °C^60^. For all heterostructure samples, 2 nm thick LAO spacer layers were deposited at 10 mTorr of O_2_ pressure at a growth rate of ~5 × 10^−2 ^Å/pulse using a single-crystalline LAO target (Shinkosa Japan). 3 nm thick TiO_2-x_ dopant layers were then deposited using a TiO_2-x_ single-crystalline target (Shinkosa Japan) at a growth rate of ~4.2 × 10^−2 ^Å/pulse in 10^−5 ^Torr of background vacuum. Finally, a 1 nm thick LAO layer was deposited under the same conditions used for the LAO spacer layer. Depositions of the spacer, dopant, and capping layers were all done at room temperature at a laser fluence of ~1.2 J/cm^2^. For all depositions, the substrate-to-target distance and the laser pulse frequency were maintained at 55 mm and 2 Hz respectively.

High-resolution Cu-*K*α X-ray diffraction spectra for both the pristine and heterostructure VO_2_ films were recorded in standard θ–2θ geometry using a Rigaku Smart Lab X-ray diffractometer. LEPTOS 7.8 software by Bruker was used to determine film thicknesses and to calculate the differential strain between pristine and heterostructure films.

Cross-sectional scanning transmission electron microscopy (STEM) imaging and energy dispersive x-ray spectroscopy (EDS) mapping were performed using TITAN Themis microscope (60–300 kV) equipped with a probe corrector and super-X four quadrant EDS detector. The high angle annual dark field (HAADF)-STEM images were acquired at an operating potential of 300 kV with a convergence angle of 24.5 mrad, 160 mm camera length, and a dwell time of 12 μs per pixel. The images were further processed with Gatan digital micrograph software. The EDS maps were acquired using Velox software under similar microscopic conditions with a dwell time of 2 μs per pixel.

Sheet resistance vs temperature measurements were performed in Van der Pauw geometry using Keithley 2450 SMU and Eurotherm 2408 PID temperature controller. Continuous temperature scanning was carried out at a rate of 4 K/min for both heating and cooling cycles.

To extract carrier density and mobility, Hall measurements for all films and heterostructures were performed using Van der Pauw geometry in a PPMS-Dynacool equipment from Quantum Design and Keithley SMU 2450 from Tektronix. For these measurements, the magnetic field was swept from 0 T to 2 T to −2 T to 0 T at a scan rate of 100 Oe/s for different temperatures ranging from 200 K to 320 K.

High-resolution hard X-ray photoelectron spectroscopy (HAXPES) measurements^61^ were carried out with an incident photon energy of 6.2 keV at the sample temperatures of 200 K (insulating phase) and 310 K (metallic phase). Binding energy calibration was carried out using a high-resolution Fermi-edge measurement on a standard Au sample. Core-level and valence-band spectra were measured using a wide acceptance angle SPECS Phoibos 225HV hemispherical electrostatic analyzer in a near-normal emission geometry. The total energy resolution was estimated to be approximately 320 meV. Preliminary HAXPES measurements and sample screening were carried out using a lab-based HAXPES instrument at Temple University equipped with a wide acceptance angle ScientaOmicron EW4000 analyzer at a total experimental energy resolution of 450 meV.

To realize the origin of the *P2* peak (at 517.5 eV) in the V *2p* core level spectra (see Fig. 5), the simulations were performed on the electron-doped VO_2_ system using LDA + DMFT Anderson impurity model calculations. Details of the calculations are presented in Supplementary Note 2 and Supplementary Fig. 16.

### Supplementary information

Supplementary Information

Peer Review File

## Data Availability

Data that support the findings of this study are available from the corresponding author upon reasonable request.
