# Supplementary material for: Modulation-doping a correlated electron insulator
Source: Nat Commun. 2023 Oct 5;14:6210. doi: 10.1038/s41467-023-41816-3 (PMC10556139; doi:10.1038/s41467-023-41816-3)
Supplement: Supplementary file 1 — Supplementary Information [file 41467_2023_41816_MOESM1_ESM.pdf]

Supplementary Information

Modulation-Doping a Correlated Electron Insulator

Debasish Mondal[1], Smruti Rekha Mahapatra[1], Abigail M Derrico[2], Rajeev Kumar Rai[3], Jay R Paudel[2], Christoph Schlueter[4], Andrei Gloskovskii[4], Rajdeep Banerjee[1], Atsushi Hariki[5], Frank M F DeGroot[6], Dipankar D Sarma[1], Awadhesh Narayan[1], Pavan Nukala[3], Alexander X Gray[2*] and Naga Phani B Aetukuri[1*]

Affiliations:

1. Solid State and Structural Chemistry Unit, Indian Institute of Science, Bengaluru, Karnataka, 560012, India
2. Department of Physics, Temple University, 1925 N. 12th St., Philadelphia, PA 19122, USA
3. Centre for Nano Science and Engineering, Indian Institute of Science, Bangalore, Karnataka, 560012, India
4. Deutsches Elektronen-Synchrotron, DESY, 22607 Hamburg, Germany
5. Department of Physics and Electronics, Graduate School of Engineering, Osaka Metropolitan University, 1-1Gakuen-cho, Nakaku, Sakai, Osaka 599-8531, Japan
6. Utrecht University, Inorganic Chemistry and Catalysis Group Universiteitsweg 99, 3584 CA Utrecht, The Netherlands

*Corresponding authors emails: phani@iisc.ac.in (N.B.A); axgray@temple.edu (A.X.G)

Table of Contents:

Supplementary Note 1: Thomas-Fermi screening length calculation for VO_2 heterostructures 3

Supplementary Note 2: The LDA+DMFT Anderson impurity model calculation V *2p* XPS spectra for electron-doped VO_2 3

Supplementary Fig. 1: Cross-sectional scanning transmission electron microscopy (STEM) images of VO_2 film and VO_2 heterostructures.......... 5

Supplementary Fig. 2: Reflection High Energy Electron Diffraction (RHEED) of VO_2 thin films 6

Supplementary Fig. 3: AFM images of VO_2 thin film and heterostructure 6

Supplementary Fig. 4: A comparison of X-ray diffractograms for VO_2 thin films and heterostructures 7

Supplementary Fig. 5: Reciprocal space maps (RSM) of VO_2 thin film and heterostructures .. 8

Supplementary Fig. 6: Temperature-dependent sheet resistance of VO_2 thin films.......... 9

Supplementary Fig. 7: Temperature-dependent sheet resistance of VO_2 thin film and heterostructures 10

Supplementary Fig. 8: Calculation of T_{MIT} from sheet resistance vs temperature curves........ 11

Supplementary Fig. 9: Temperature-dependent sheet resistance of VO_2 heterostructures and controls.......... 12

Supplementary Fig. 10: Carrier density and carrier mobility for 7.5 nm VO_2 thin films and heterostructures 13

Supplementary Fig. 11: Binding energy calibration of VO_2 spectra across the MIT.......... 14

Supplementary Fig. 12: Summary of Binding energy changes for modulation-doped VO_2 15

Supplementary Fig. 13: Evolution of *P1* peak in metallic and insulating VO_2 heterostructures 16

Supplementary Fig. 14: HAXPES spectra of V $2p_{3/2}$ 17

Supplementary Fig. 15: HAXPES spectra of La *3d* and Al *1s* 18

Supplementary Fig. 16: V *2p* XPS spectra of electron-doped VO_2 calculated by the LDA+DMFT-Impurity method.......... 19

Supplementary Fig. 17: Valence band spectra in the insulating state for VO_2 heterostructures and thin films 20

Supplementary Fig. 18: Comparison of X-ray diffractograms between VO_2 thin film and heterostructure in both the phases of VO_2 21

Supplementary Table 1: Out-of-plane lattice parameter (c_R) and T_{MIT} for VO_2 films and heterostructures 22

Supplementary References.......... 23

Supplementary Note 1: Thomas-Fermi screening length calculation for VO_2 heterostructures

Thomas-Fermi screening length (L) can be calculated as follows

$$L = \sqrt{\frac{K\varepsilon_0 T k_B}{e^2 n_e}}$$

where critical carrier density, $n_e \approx \left(\frac{0.25}{a_B}\right)^3$ and K, ε_0, k_B, T, e, a_B are the dielectric constant ($\approx$ 36)[1,2] of VO_2, free space permittivity ($\approx 8.854\text{x}10^{-12}$ F/m), Boltzmann constant ($\approx 1.380\text{x}10^{-23}$ m^2 kg s^{-2} K^{-1}), temperature of VO_2, electronic charge ($\approx 1.602\text{x}10^{-19}$ C) and effective Bohr radius respectively. Effective Bohr radius (a_B) can be calculated as follows

$$a_B = \frac{h^2 K \varepsilon_0}{\pi m^* e^2}$$

where h is the Planck's constant ($\approx 6.626\text{x}10^{-34}$ m^2kg/s) effective mass of electron in VO_2, $m^* \approx 3.5 m_e$.[2,3] By plugging these above values and keeping T $\approx$ 300 K, calculated Thomas-Fermi screening length (L) is ~0.73 nm.

Supplementary Note 2: The LDA+DMFT Anderson impurity model calculation V *2p* XPS spectra for electron-doped VO_2

We here present a computational simulation of V *2p* X-ray photoemission spectroscopy (XPS) of metallic VO_2 samples using the local density approximation (LDA) + dynamical mean-field theory (DMFT) method. Our computational implementation is given in References [4] and [5]. First, a standard LDA+DMFT calculation is performed for the experimental structure of VO_2 in a high-temperature metallic phase. The LDA bands obtained using the Wien2K package[6] are subsequently mapped onto the tight-binding model spanning the V *3d* and O *2p* states with the wien2wannier and wannier90 packages.[7,8] Then, the tight-binding model is augmented with the local electron-electron interaction within the V *3d* shell. We chose Hubbard interaction U=6.0 eV and Hund's interaction J=1.0 eV, consulting with previous ab-initio and spectroscopy studies for VO_2.[9,10] The continuous-time quantum Monte-Carlo impurity solver with the strong-coupling formalism was

used to solve the auxiliary Anderson impurity model (AIM) in the DMFT self-consistent equation. After the convergence is reached, the self-energy in the real-frequency domain obtained via the maximum entropy method was used to compute the valence spectral functions and the hybridization densities. The V *2p* core-level XPS spectra are calculated using the AIM with the LDA+DMFT hybridization densities, where the V *2p* core orbitals and their interaction with the V *3d* electrons are considered explicitly. The V *2p*-*3d* interaction parameters are determined following References [4] and [5].

The configuration-interaction AIM solver implementing the intra-atomic full-multiplet interaction was employed to compute the V *2p* XPS spectral intensities. The LDA+DMFT method was successfully applied to analyze the 2p core-level spectra of V_2O_3 and other early to late 3d transition-metal oxides.[4,11,12] To examine the 2p XPS spectral change by electron doping, we performed the LDA+DMFT calculations for the undoped and electron-doped VO_2. The electron doping was simulated by changing the total number of the valence electrons in the V *3d* - O *2p* lattice model constructed from the LDA for the undoped VO_2 system as mentioned above. Thus, though the DMFT self-consistency condition was updated for the electron-doped system, a lattice relaxation or a disorder effect by the doping was not considered in the present simulation. Though the LDA+DMFT method is known to suffer from the lack of the nonlocal inter-site (V-V) self-energy in describing the dimerized insulating ground state of VO_2, it provides a reasonable description for its correlated metallic phase as discussed in Reference [13].

In Supplementary Fig. 16, the LDA+DMFT spectrum of the undoped VO_2 excellently reproduces the present and previously reported experimental V *2p* XPS data of the metallic phase, including the low-binding-energy feature around 514.5 eV (*P1* feature) due to a metallic screening. Given the good agreement in the undoped VO_2, we calculated the V *2p* XPS of the electron doped VO_2 (0.2 el. per V atom to the formal V^{4+} valence count). The *P1* feature is slightly suppressed in the electron-doped VO_2 compared with the undoped one, in agreement with the experiment. Importantly, the *P2* peak at 517.5 eV is developed in the electron-doped VO_2 spectrum. The binding energy as well as the intensity of the *P2* peak with respect to the V *2p*3/2 main peak matches nicely with the experimental data in the main text, suggesting that the *P2* peak is intrinsic in the electron-doped metallic VO_2 samples. Though the presence of the *P2* peak in the electron-doped metallic VO_2 samples was supported in the LDA+DMFT simulation, it may be

counterintuitive to have a high-binding-energy peak with creating lower-valence V^{3+} species by the electron doping. However, a material specific consideration is often needed to interpret the 2p core-level XPS line features, especially in transition-metal oxides.

First, the V $2p_{3/2}$ main-line shape is sensitive to the V-O covalency that is specific for the V valence state. This is because the main line is composed of the V-O bonding final states and consequently even a small change of the V-O hybridization causes a binding-energy shift and an intensity modulation in the V *2p* main-line features.[11,14] The V-O covalency for the V^{3+} (d^2) state is weaker than V^{4+} (d^1) one, and thus the binding energy of V^{3+} is shifted to a higher-binding-energy due to a weaker bonding and anti-bonding splitting in the XPS final states. This would explain the appearance of the satellite feature at a high-binding-energy side of the V $2p_{3/2}$ line with electron doping, although the *P2* and V $2p_{3/2}$ mainline peaks do not represent the specific ionic valence states due to a complex chemical bond formation as well as a quantum mechanical interference effect in the 2p XPS final states. Furthermore, a strong V *2p-3d* core-valence multiplet interaction is present in the XPS final states and affects the line shape. Note that the V^{3+} (d^2) atomic state is more multiplet rich than the V^{4+} (d^1) one. In Supplementary Fig. 16, we calculated the V *2p* XPS spectrum without the V *2p-3d* multiplet interaction term in the LDA+DMFT AIM Hamiltonian.[4,5] It demonstrates that the *P2* intensity is enhanced due to the presence of the V *2p-3d* multiplet interaction and indicates the V *2p-3d* multiplet admixes the spectral features of the different valence states in the V $2p_{3/2}$ core-level line.

Supplementary Fig. 1: Cross-sectional scanning transmission electron microscopy (STEM) images of VO_2 film and VO_2 heterostructures.

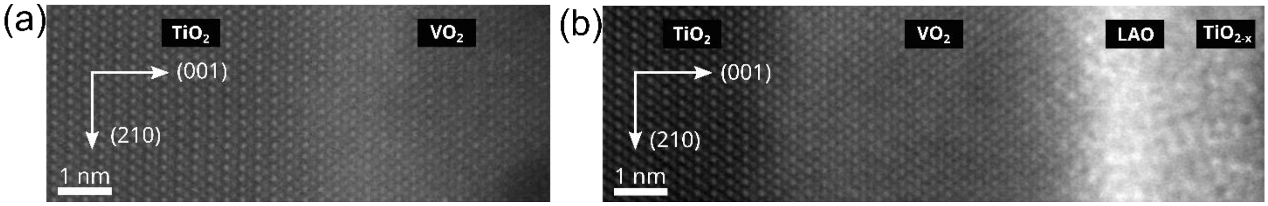

Supplementary Fig. 1. Room temperature STEM images of (a) 6 nm VO_2 on TiO_2 (001) substrate and (b) 6 nm VO_2/ 2 nm LAO/ 3 nm TiO_{2-x}/1 nm LAO deposited on TiO_2 (001) substrate. In both cases, VO_2 is coherently strained to the TiO_2 (001) substrate.

Supplementary Fig. 2: Reflection High Energy Electron Diffraction (RHEED) of VO_2 thin films

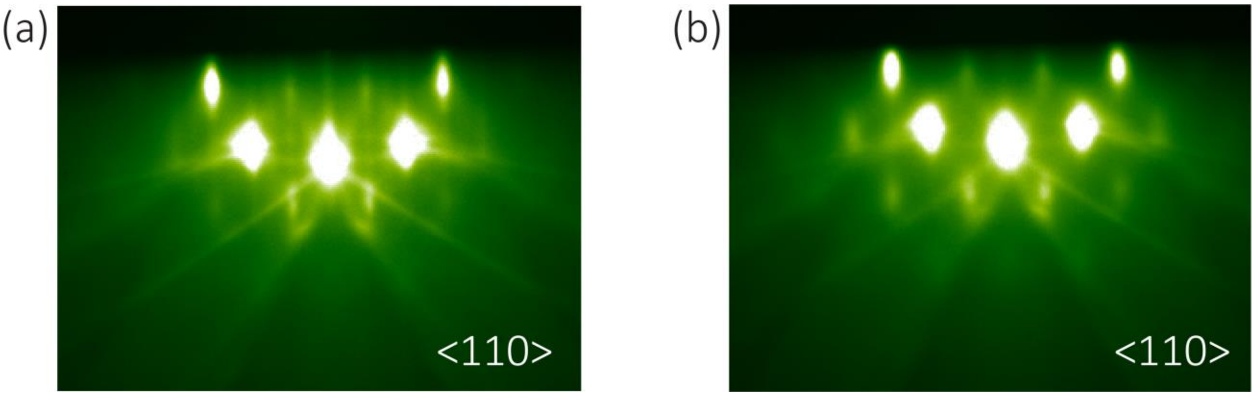

Supplementary Fig. 2. RHEED pattern of VO_2 thin films on TiO_2 (001) substrate along the <110> direction for two different thicknesses of (a) 1.5 nm and (b) 9.5 nm. These patterns were captured at 425 °C just after the VO_2 deposition. RHEED patterns are indicative of single-crystalline VO_2 films with smooth film surfaces.

Supplementary Fig. 3: AFM images of VO_2 thin film and heterostructure

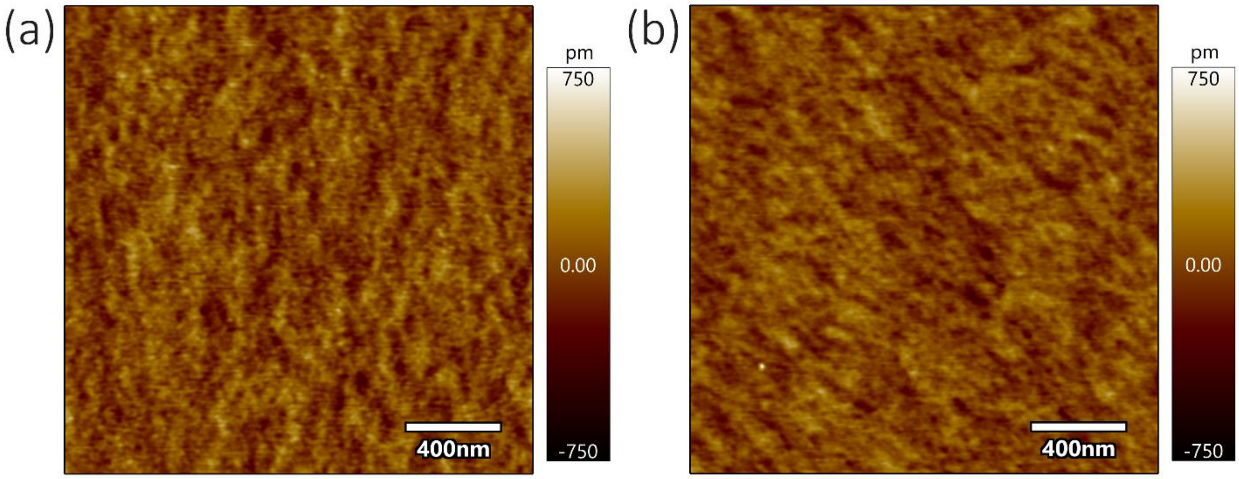

Supplementary Fig. 3. AFM images of (a) 9.5 nm VO_2 film and (b) 9.5 nm VO_2 heterostructure on TiO_2 (001) substrate show smooth 2D surfaces with a root mean-square roughness in the range of 80-100 pm. The images were taken in the tapping mode (AC air topography) using an Asylum Cypher ES AFM.

Supplementary Fig. 4: A comparison of X-ray diffractograms for VO_2 thin films and heterostructures

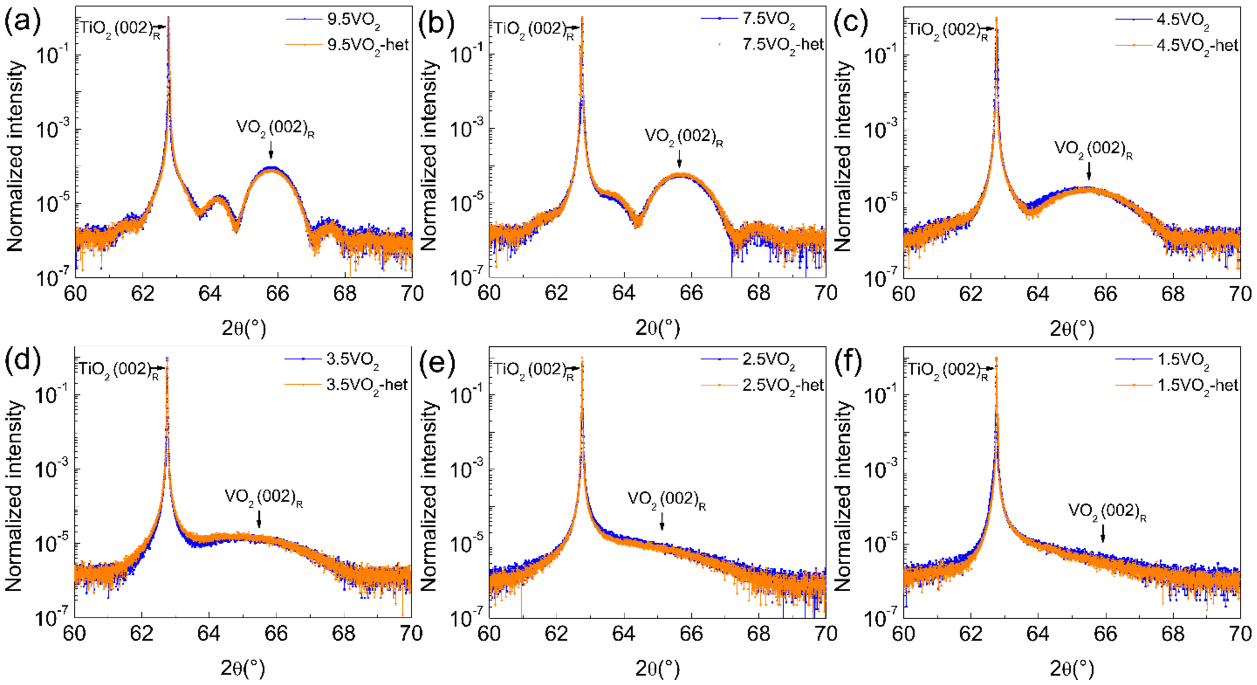

Supplementary Fig. 4. Comparison of high-resolution θ-2θ X-ray diffractograms of VO_2 (001) thin films (blue) and heterostructures (orange) at different VO_2 thicknesses of (a) 9.5 nm, (b) 7.5 nm, (c) 4.5 nm, (d) 3.5 nm, (e) 2.5 nm, and (f) 1.5 nm. We showed in Fig. 2a of the main manuscript that there is no measurable change in the VO_2 lattice parameter, C_R, between VO_2 thin films and heterostructures. To further confirm this, we performed and compared high-resolution X-ray diffractograms for thin films and heterostructures with identical VO_2 thickness. Clearly, there is excellent overlap of the two diffractograms, including thickness fringes, for all thicknesses measured. This is a clear indication that depositing heterostructures on ultra-thin VO_2 films did not affect the lattice parameters of VO_2.

.

Supplementary Fig. 5: Reciprocal space maps (RSM) of VO_2 thin film and heterostructures

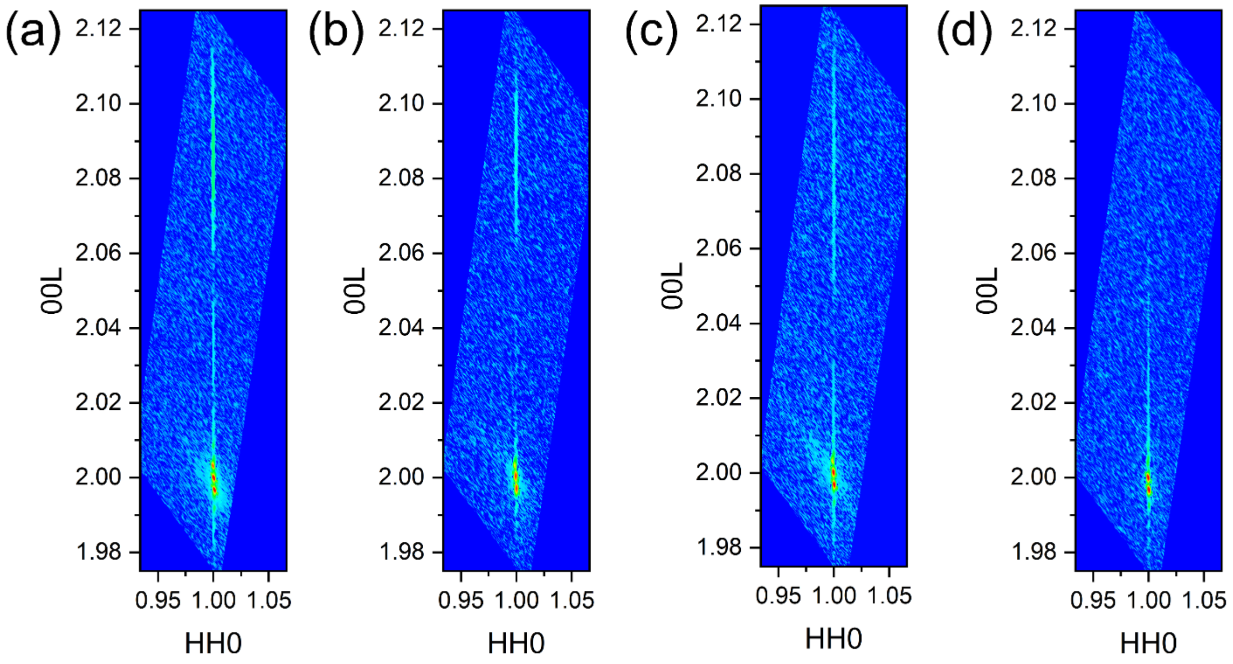

Supplementary Fig. 5. Asymmetrical RSM images around (112) plane for (a) 9.5 nm VO_2 thin film, and VO_2 heterostructures with VO_2 thicknesses of (b) 9.5 nm (c) 6.5 nm and (d) 1.5 nm. The vertical and horizontal axis of all the plots is scaled relative to the miller indices of the TiO_2 (001) substrate. The RSM data presented here is further evidence that there are no measurable changes to the unit cell volume across all heterostructures and that all films are coherently strained to the TiO_2 (001) substrate.

Supplementary Fig. 6: Temperature-dependent sheet resistance of VO_2 thin films

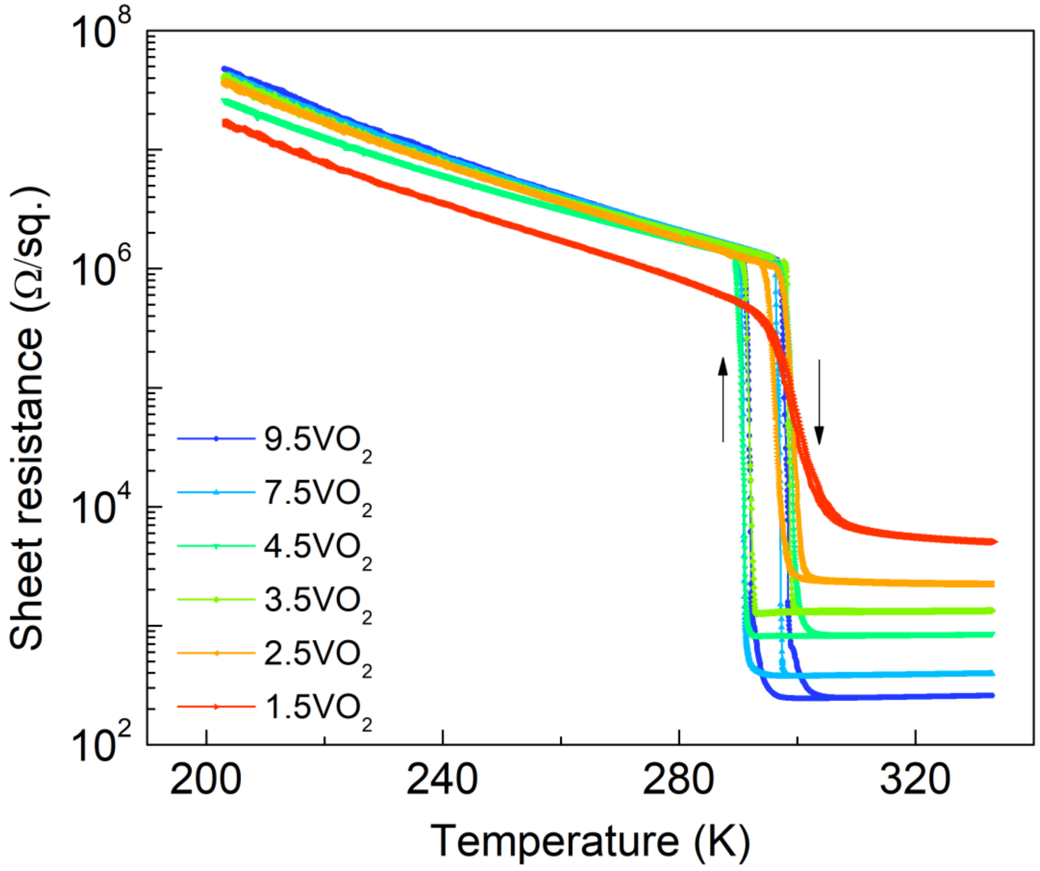

Supplementary Fig. 6. Temperature-dependent sheet resistance of VO_2 thin films with varying VO_2 thicknesses as mentioned in the legends. For nomenclature simplicity, VO_2 thin films are written as tVO_2 where 't' is the thickness of VO_2. For example, 9.5VO_2 corresponds to a 9.5 nm thick VO_2 thin film. The increase in metallic state resistance as the thickness of VO_2 film is decreased is suggestive of interfacial scattering. This also shows all the films, except 1.5VO_2 and 2.5VO_2, have nearly the same transition temperature (~295 K) which again confirms the reduction in transition temperature in VO_2 heterostructures is entirely after the formation of the heterostructure. The slight increase in transition temperature for the 1.5VO_2 and 2.5VO_2 film is attributed to be due to titanium interdiffusion at the VO_2/TiO_2(substrate) interface.[15,16]

Supplementary Fig. 7: Temperature-dependent sheet resistance of VO_2 thin film and heterostructures

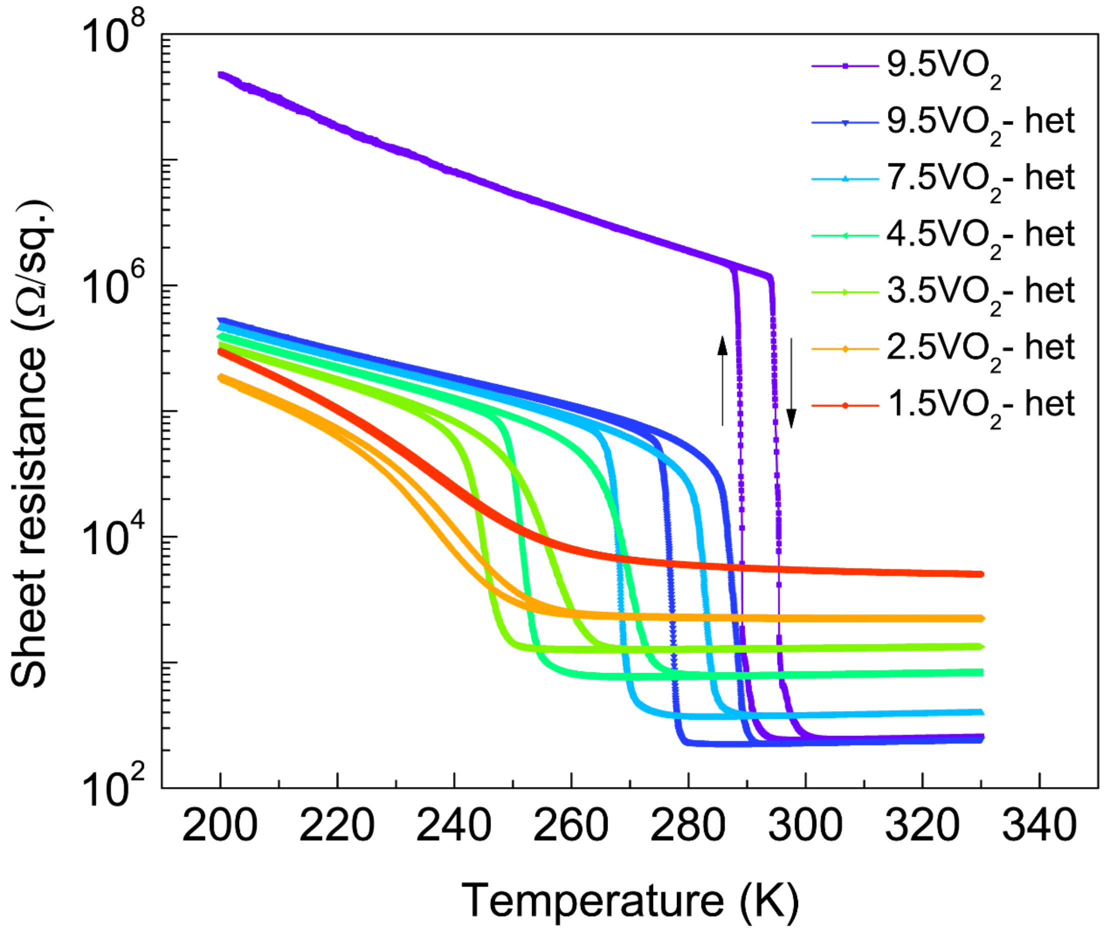

Supplementary Fig. 7. Temperature-dependent sheet resistance of 9.5 nm VO_2 thin film and VO_2 heterostructures with varying VO_2 thicknesses, t. For nomenclature simplicity, we distinguish VO_2 thin films and heterostructures with a VO_2 thickness of 't' as tVO_2 and tVO_2-het, respectively. For example, 9.5VO_2-het corresponds to a heterostructure with 9.5 nm thick VO_2. The sheet resistance data in this figure corresponds to the normalized resistance presented in Fig. 2b of the main text. The increase in metallic state resistance as the thickness of VO_2 film is decreased is suggestive of interfacial scattering. Importantly, it can be seen that the metallic state resistance is identical for the 9.5 nm VO_2 heterostructure and 9.5 nm VO_2 thin film. This is also an indirect indication of the sharp interfaces across the heterostructure layers; Rough interfaces could lead to increased interfacial scattering and a higher metallic state resistance for the 9.5 nm VO_2 heterostructure in comparison with the 9.5 nm VO_2 thin film.

Supplementary Fig. 8: Calculation of T_{MIT} from sheet resistance vs temperature curves

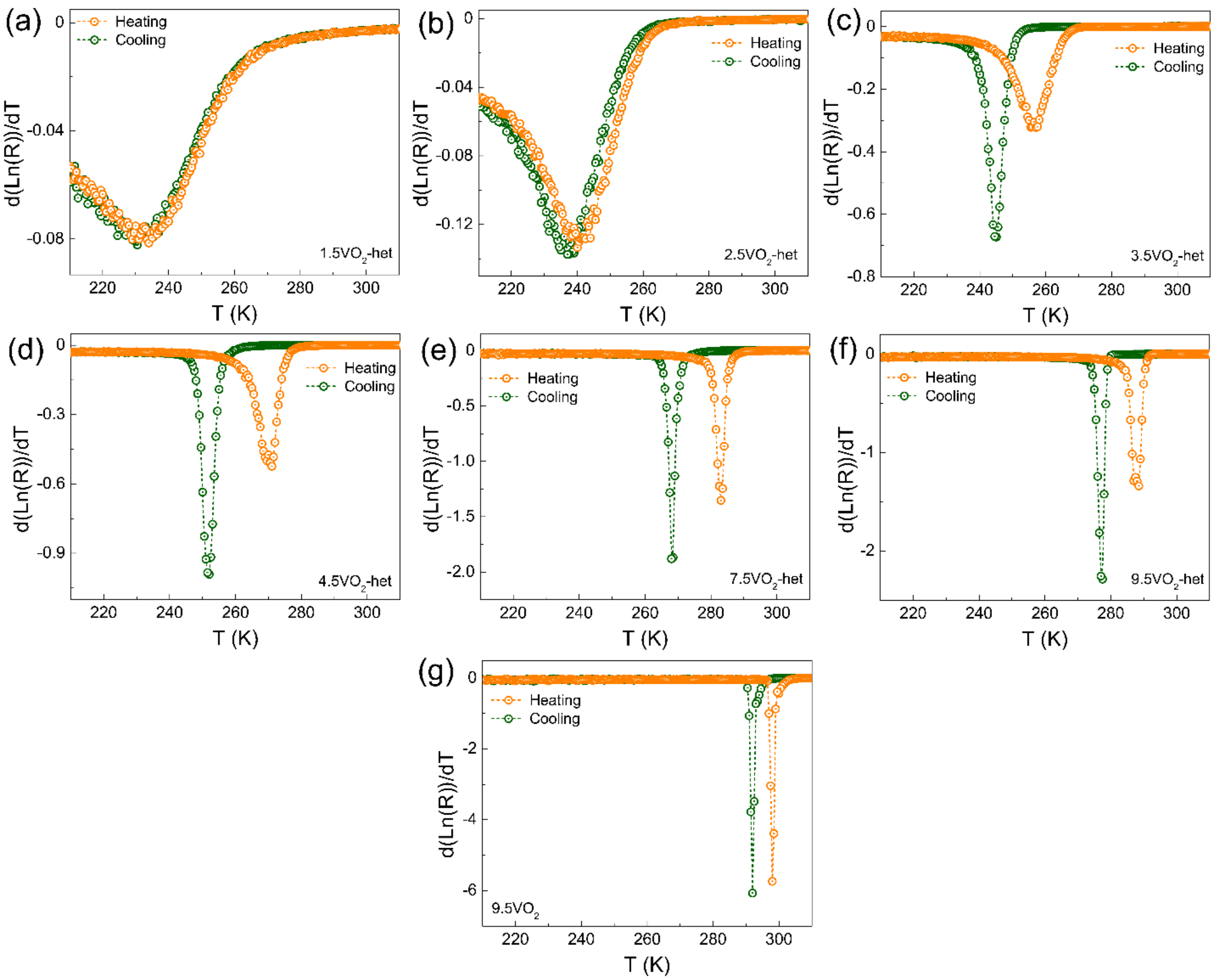

Supplementary Fig. 8. d(ln(R))/dT plots for both the heating and cooling cycles for VO_2 heterostructures with VO_2 thicknesses of (a) 1.5 nm, (b) 2.5 nm, (c) 3.5 nm, (d) 4.5 nm, (e) 7.5 nm, (f) 9.5 nm, and (g) for a 9.5 nm VO_2 thin film. Here, ln(R) is the natural log of the temperature-dependent sheet resistance of the films as shown in Supplementary Fig. 7 and d/dT is the derivative operator with respect to temperature, T. Peak positions of the d(ln(R))/dT plots correspond to the transition temperature for the respective thermal cycle. All the transition temperatures discussed in the main text are the average transition temperature of the transition temperature for the heating and cooling cycle given by $T_{MIT} = ((T_{MIT})_{heating} + (T_{MIT})_{cooling})/2$. The estimated T_{MIT} are: 233 K for 1.5 nm VO_2; 237 K for 2.5 nm VO_2; 250 K for 3.5 nm VO_2; 260 K for 4.5 nm VO_2; 275 K for 7.5 nm VO_2; 282 K for 9.5 nm VO_2 in VO_2 heterostructures. By comparison, the T_{MIT} is 295 K for 9.5 nm VO_2 thin film.

Supplementary Fig. 9: Temperature-dependent sheet resistance of VO_2 heterostructures and controls

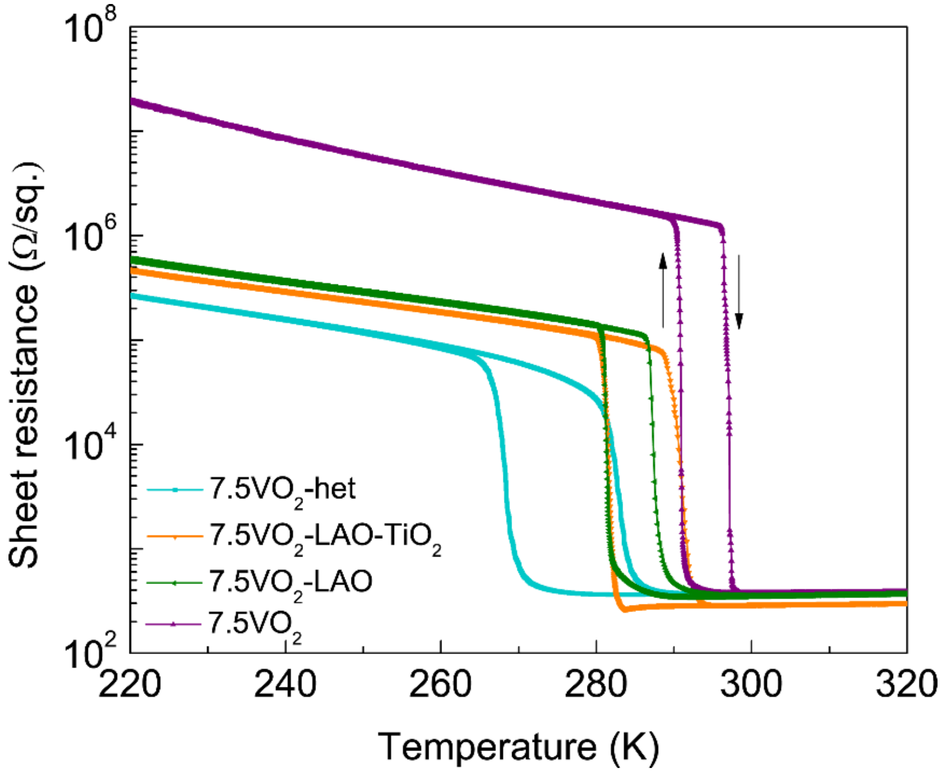

Supplementary Fig. 9. A comparison of temperature-dependent sheet resistance characteristics of modulation-doped 7.5 nm VO_2 heterostructure (7.5VO_2-het) and 7.5 nm VO_2 thin film (7.5VO_2) with various VO_2 heterostructure 'controls'. The control heterostructures include 7.5 nm VO_2 thin film capped with 2 nm LAO (7.5VO_2-LAO) and a 7.5 nm VO_2 heterostructure with a near stoichiometric TiO_2 deposited at 10 mTorr of oxygen pressure with a 1 nm LAO capping layer over TiO_2 (7.5VO_2-LAO-TiO_2). The number prefixed with each of the layers represent the respective film thickness in nm. For the 7.5VO_2-LAO heterostructure, there is a reduction in resistivity in the insulating phase and a small but observable reduction in transition temperature (T_{MIT}) ~8 K compared to the T_{MIT} of VO_2 thin film. However, for the modulation-doped heterostructure, (7.5VO_2-het), there is a further reduction in resistivity with a decrease in T_{MIT} of ~20 K compared to the T_{MIT} of VO_2 thin film. For the heterostructure where TiO_2 is near stoichiometric (7.5VO_2-LAO-TiO_2), the reduction in T_{MIT} is also ~8 K, similar to the reduction in T_{MIT} for 7.5VO_2-LAO. Clearly, a majority of the reduction in T_{MIT} comes from the charge transfer from the TiO_{2-x} dopant layer.

Supplementary Fig. 10: Carrier density and carrier mobility for 7.5 nm VO_2 thin films and heterostructures

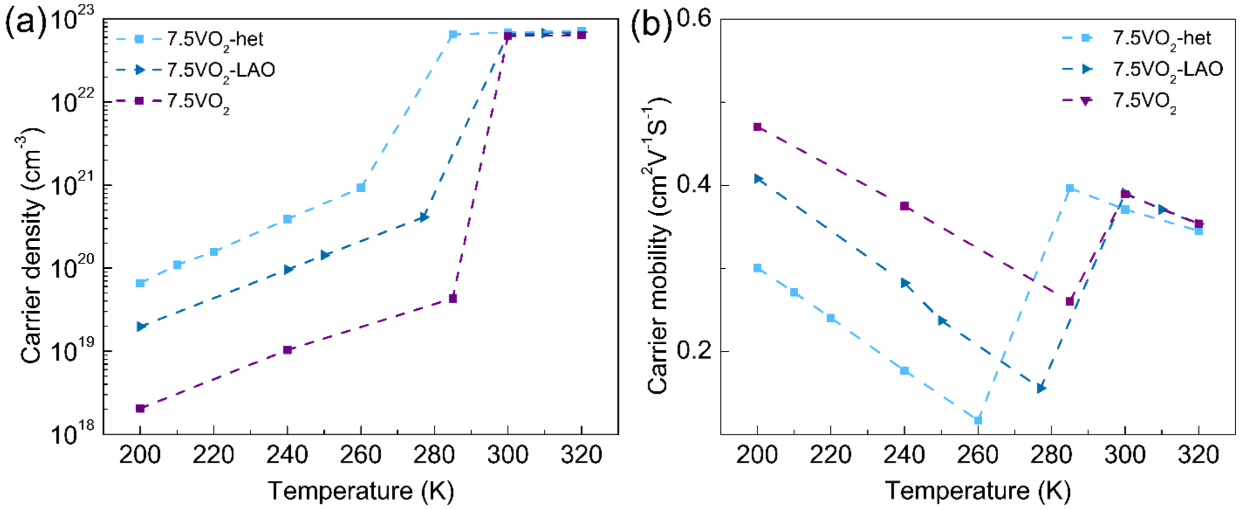

Supplementary Fig. 10. Plots of temperature-dependent (a) carrier densities and (b) carrier mobilities for 7.5 nm VO_2 heterostructure (7.5VO_2-het), 7.5 nm VO_2 thin film (7.5VO_2) and 7.5 nm VO_2 capped with 2 nm LAO (7.5VO_2-LAO). For the 7.5VO_2-LAO, there is a noticeable increase in carrier density (and a decreased in carrier mobility) compared to 7.5 nm VO_2 thin film, but this is less than the carrier density observed in the comparable modulation-doped heterostructure (7.5VO_2-het).

Supplementary Fig. 11: Binding energy calibration of VO_2 spectra across the MIT

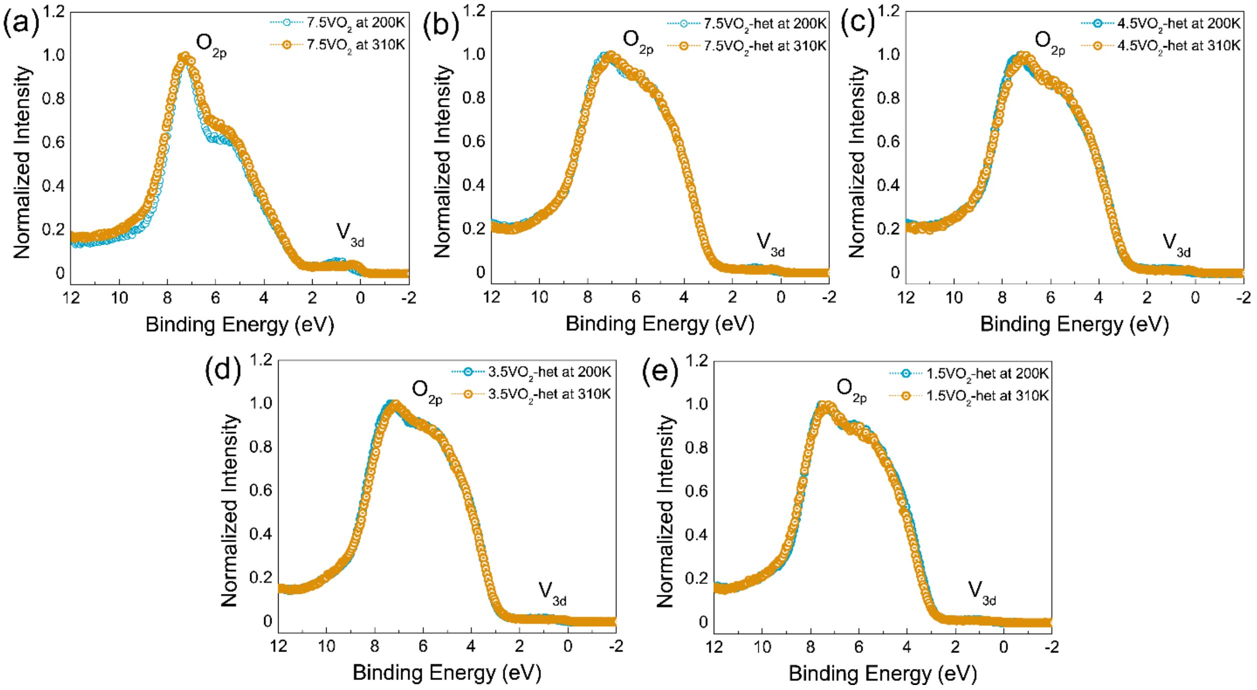

Supplementary Fig. 11. Binding energy calibration for the insulating state spectra. We noticed charging effects in some of the insulating state HAXPES spectra. In order to correct for charging effects, we used O *2p* binding energy across insulating and metallic states of the samples as an internal reference. We note that metallic state spectra are unaffected by charging affects and several previous studies showed that the binding energy of O *2p* spectra does not change across the MIT[17–20]. Further, no changes to the O *2p* contributions from the LAO and TiO_2 layers are expected across the MIT in VO_2. The valence band spectra for (a) 7.5 nm VO_2 film and VO_2 heterostructures with VO_2 thicknesses of (b) 7.5 nm, (c) 4.5 nm, (d) 3.5 nm, and (e) 1.5 nm are shown for both the metallic phase (at 310 K) and the insulating phase (at 200 K) after binding energy correction. All the V *2p* spectra shown in the main text are based on this calibration.

Supplementary Fig. 12: Summary of Binding energy changes for modulation-doped VO_2

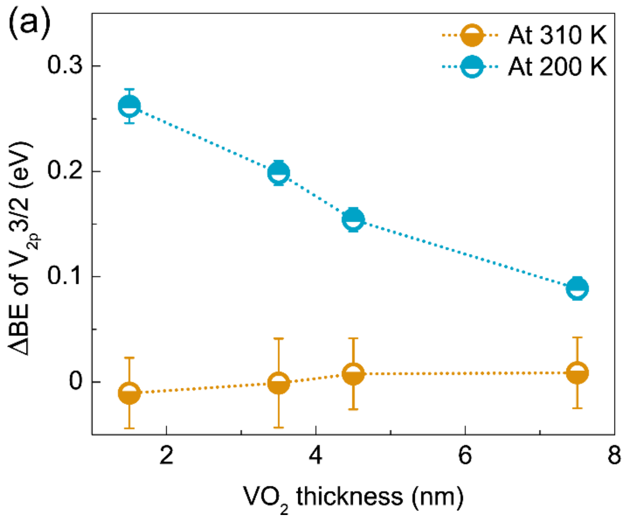

Supplementary Fig. 12. The change in binding energy of the V *2p*$_{3/2}$ peak for VO_2 heterostructures with respect to its binding energy in VO_2 thin films (ΔBE) for various thicknesses of VO_2 in heterostructures. There is no binding energy change in the metallic state (orange, at 310 K), while there is a binding energy increase for the same set of samples in the insulating state (blue, at 200 K). This is suggestive of band-bending leading to modulation-doping. Error bars represent the combined error of the energy stability of the beamline, which is typically +/-10 meV, and the error from peak fitting, which is governed by your signal-to-noise ratio.

Supplementary Fig. 13: Evolution of *P1* peak in metallic and insulating VO_2 heterostructures

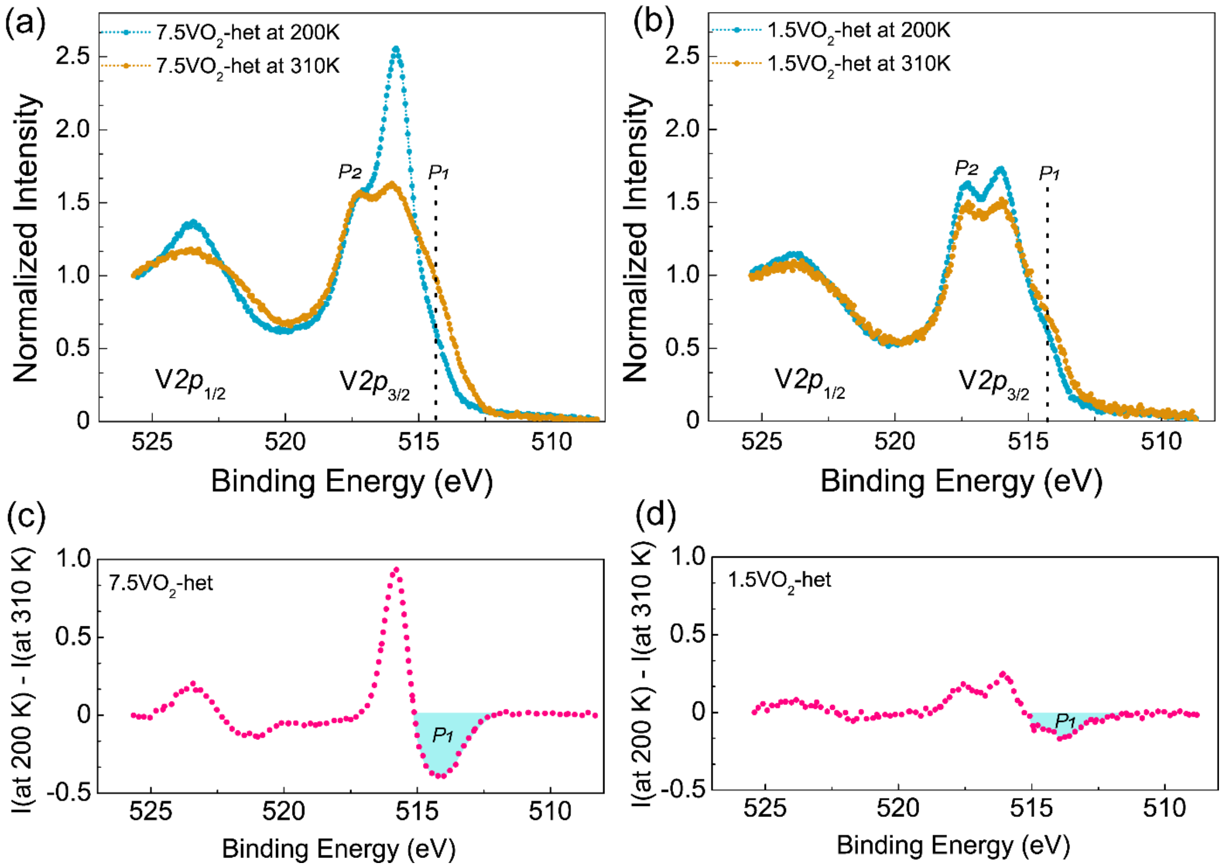

Supplementary Fig. 13. V *2p* core level spectra for (a) 7.5 nm and (b) 1.5 nm VO_2 heterostructures in the metallic (at 310 K) and insulating (at 200K) states normalized to the V *2p* area under the curve. Corresponding intensity difference plots in (c) show that the *P1* peak (at ~514.5 eV) is prominent in the metallic phase for the 7.5 nm VO_2 heterostructure (negative intensity difference) while for the 1.5 nm heterostructure in (d), the intensity difference decreases suggesting an increase in the *P1* intensity for the insulating state spectrum for the 1.5 nm VO_2 heterostructure.

Supplementary Fig. 14: HAXPES spectra of V *2p*3/2

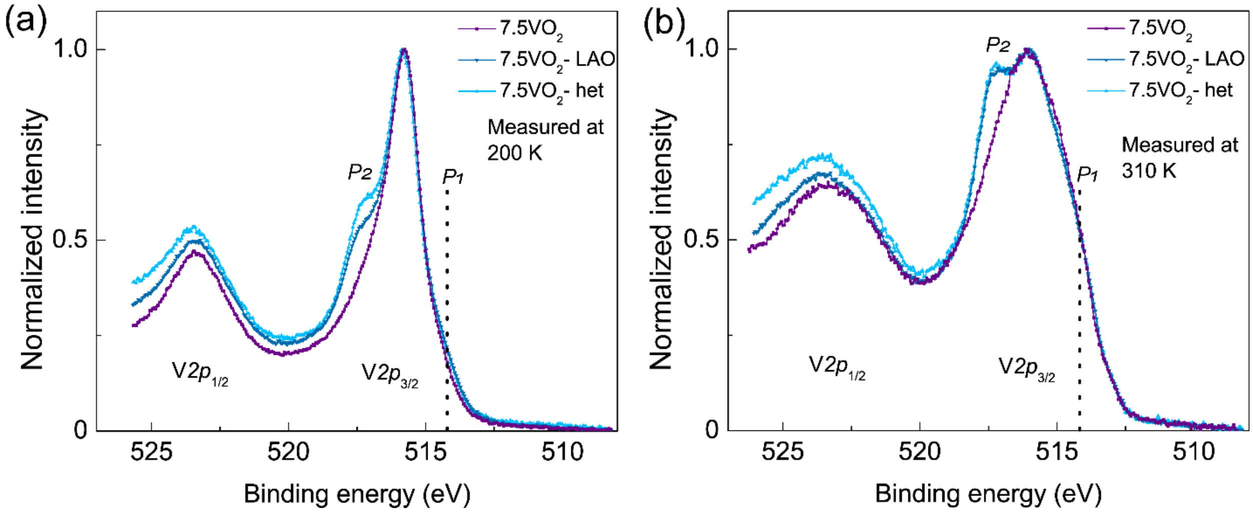

Supplementary Fig. 14. A comparison of V *2p* core-level spectra of 7.5 nm modulation doped VO_2 heterostructure, 7.5 nm VO_2 with 2 nm LAO capping layer and 7.5 nm VO_2 thin film in (a) the insulating (200 K) and (b) the metallic states (310 K). The additional peak *P2* is observed after the deposition of LAO. However, the change in the peak *P1* in the insulating state spectra is not significant.

Supplementary Fig. 15: HAXPES spectra of La *3d* and Al *1s*

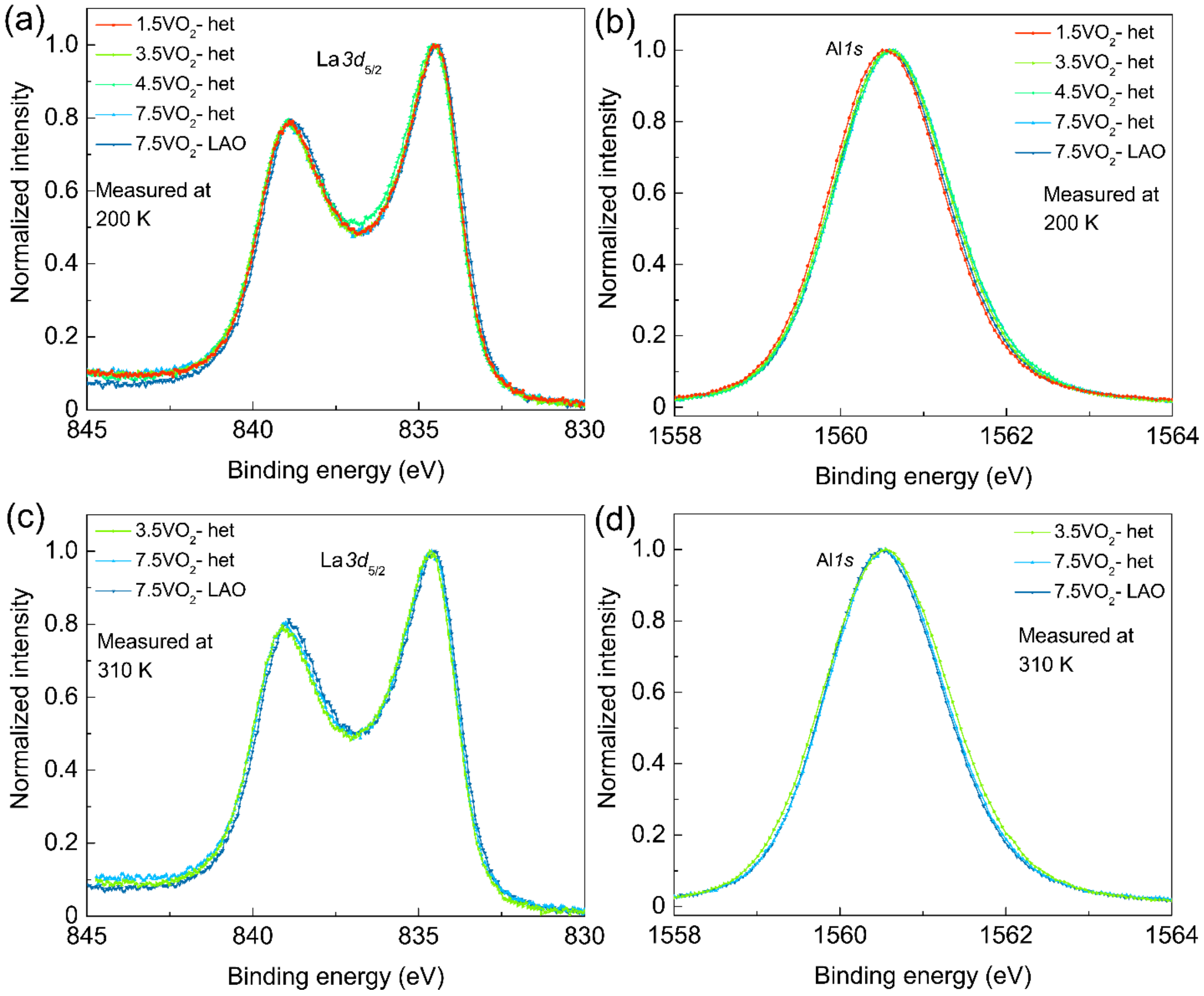

Supplementary Fig. 15. A comparison of core level HAXPES spectra of modulation-doped VO_2 heterostructures of (a) La $3d_{5/2}$ and (b) Al 1s at 200 K, and (c) La $3d_{5/2}$ (d) Al 1s at 310 K. In the insulating state (at 200 K), the spectra were collected for VO_2 heterostructures corresponding to all VO_2 thicknesses used in this study. In the metallic state (at 310 K), the spectra were collected for 3.5 nm and 7.5 nm VO_2 heterostructures and for 7.5VO_2/2LAO heterostructure. No significant changes to the La and Al core levels were observed.

Supplementary Fig. 16: V *2p* XPS spectra of electron-doped VO_2 calculated by the LDA+DMFT-Impurity method

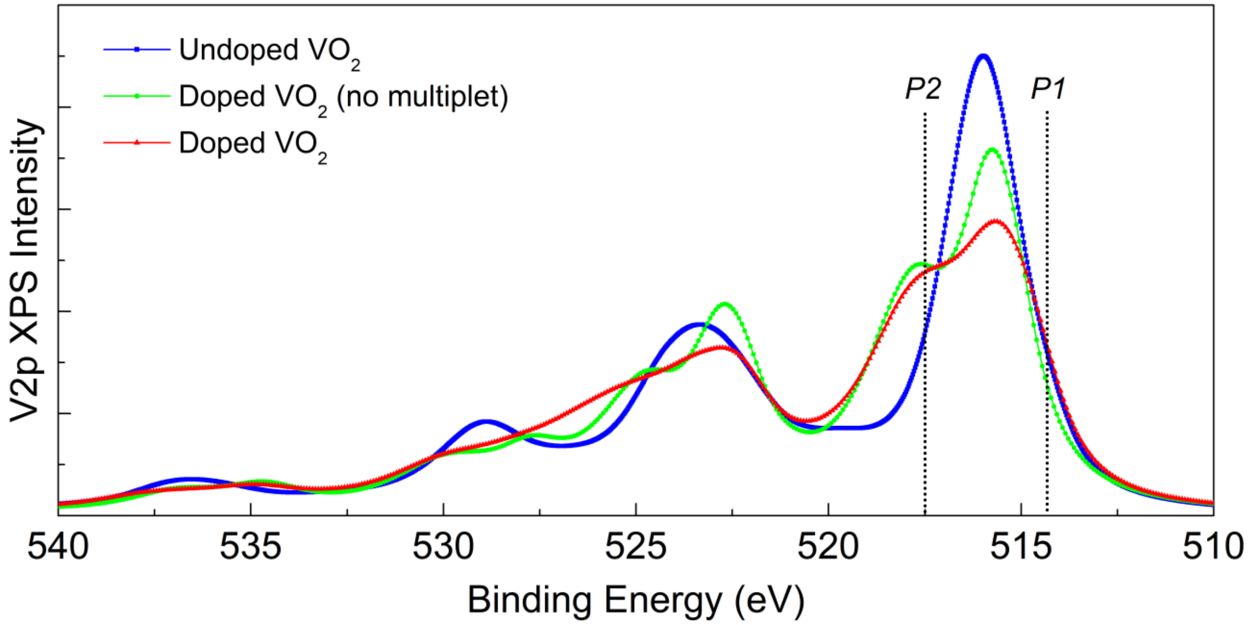

Supplementary Fig. 16. V *2p* XPS spectra calculated by the LDA+DMFT Anderson Impurity method for undoped (stochiometric) VO_2 (blue), and electron-doped VO_2 (red). The V *2p* XPS spectrum of the electron-doped VO_2 without the *2p*-*3d* core-valence multiplet interaction is also shown (green). The labels *P1* and *P2* are referred to as the non-local screening feature and an unusual feature at a higher binding energy of the main line peak respectively as observed in HAXPES measurements (see Fig. 5 in main text). However, based on the LDA+DMFT Anderson Impurity simulation the *P2* peak is assigned as a satellite peak induced by electron doping in VO_2 as described in Supplementary Note 2.

Supplementary Fig. 17: Valence band spectra in the insulating state for VO_2 heterostructures and thin films

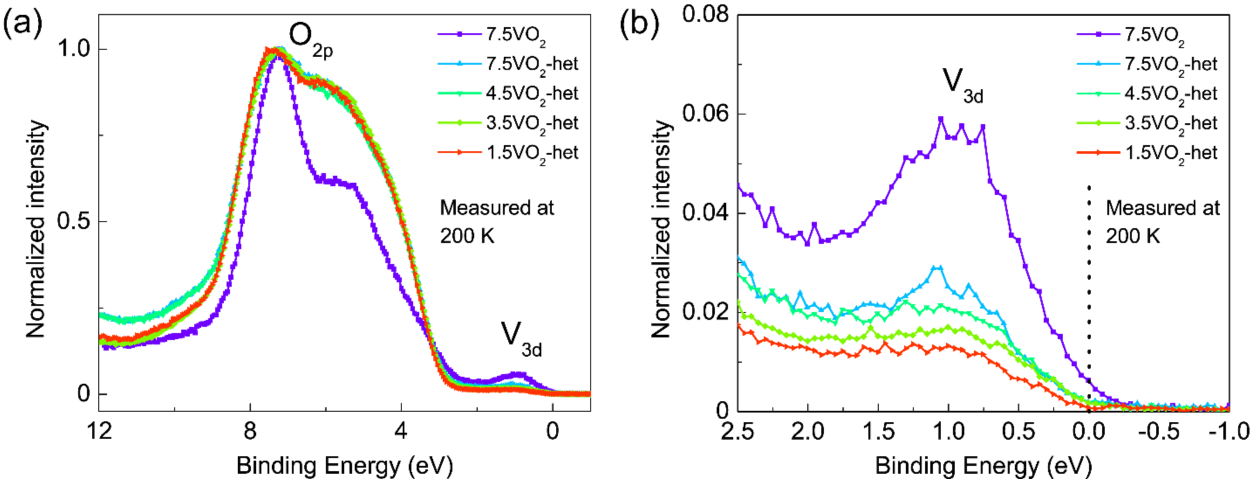

Supplementary Fig. 17. (a) Valence band spectra of O *2p* and V *3d* and, (b) V *3d* spectra (zoom in of Supplementary Fig. 17 (a) measured at 200 K are shown here after the binding energy correction as discussed in Supplementary Fig. 11. The V *3d* peak position at ~0.9 eV is in good agreement with existing literature.[17–20] The vertical dotted line at 0 eV corresponds to the Fermi level.

Supplementary Fig. 18: Comparison of X-ray diffractograms between VO_2 thin film and heterostructure in both the phases of VO_2

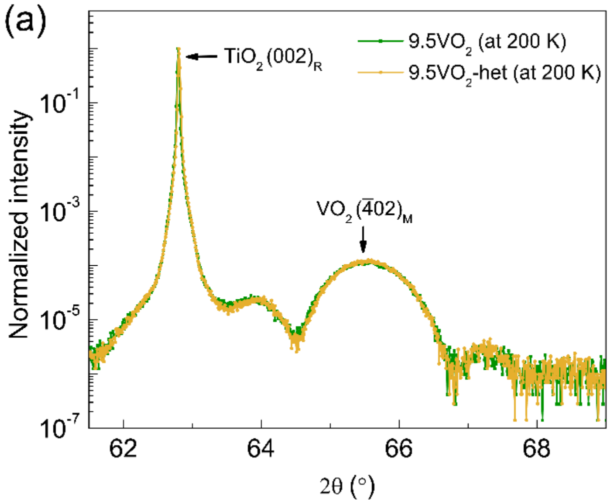

Supplementary Fig. 18. A comparison of XRD diffractograms of 9.5VO_2 and 9.5VO_2-het measured at (a) 200 K and (b) 320 K. An excellent overlap of Bragg reflections along with thickness fringes can be seen. This clearly suggests that there are no measurable structural changes induced due to heterostructuring.

Supplementary Table 1: Out-of-plane lattice parameter (c_R) and T_{MIT} for VO_2 films and heterostructures

VO_2 thickness (nm)	Extracted out-of-plane lattice parameter (C_R) from XRD fitting (pm)		ΔC_R = ($C_{R\text{-het}}$ - $C_{R\text{-film}}$) (pm)	Transition temperature (T_{MIT}) (K)		ΔT_{MIT} = ($T_{MIT\text{-het}}$ - $T_{MIT\text{-film}}$) (K)
	VO_2 film ($C_{R\text{-film}}$)	VO_2 heterostructure ($C_{R\text{-het}}$)		VO_2 film ($T_{MIT\text{-film}}$)	VO_2 heterostructure ($T_{MIT\text{-het}}$)	
9.5	283.33	283.34	0.01	295	282	-13
7.5	283.24	283.25	0.01	295	275	-20
4.5	283.23	283.24	0.01	295	260	-35
3.5	284.11	284.11	0	295	250	-45
2.5	284.28	284.28	0	297	237	-60
1.5	284.29	284.29	0	299	233	-66

Supplementary Table 1. A comparison of out-of-plane lattice parameters (c_R) and T_{MIT} between VO_2 film and VO_2 heterostructures. LEPTOS 7.8 from Bruker was used to extract the lattice parameters using a pseudo-voigt function for all the films and heterostructures. Based on θ-2θ X-ray diffractograms in Fig. 2a, there is an apparent downshift in the position of the VO_2 (002) with decreasing film thickness. We note that this downshift in the angular position of the VO_2 (002) reflection with decreasing film thickness is inconsistent with strain relaxation which should downshift the VO_2 (002) reflection's angular position with increasing thickness. This is because the in-plane lattice parameters of TiO_2 (001) (a_{TiO2} = 4.59 Å) are greater than bulk VO_2 (001) (a_{VO2} = 4.55 Å). Strain relaxation, if any, (possible with increasing thickness) should decrease out-of-plane compression and increase the out-of-plane lattice parameter (leading to a downshift in the angular position of VO_2 (002) reflection) with increasing VO_2 thickness. Therefore, we hypothesized that the apparent downshift may be due to an increased contribution from the TiO_2 (002) substrate reflection to the VO_2 (002) reflection in heterostructures with thinner VO_2. However, at lower film thicknesses, the total intensity of the VO_2 film reflection was too weak to accurately disentangle the intensity contributions from the TiO_2 substrate reflection and the VO_2 reflection. Therefore, we deposited films and heterostructures of the same thickness and compared the changes in the out-of-plane lattice parameters and the T_{MIT} across the film and heterostructure with the same VO_2 thickness. It can be seen that the out-of-plane lattice parameters for both the

film and heterostructures are nearly identical for the same thickness of VO_2. Hence the differential out-of-plane lattice parameters (ΔC_R) are independent of the VO_2 thickness suggesting there are no changes in strain due to the formation of heterostructures. The errors in the calculation of the lattice parameter if any are due to the broadening of the VO_2 film's reflection as the thickness is decreased. Our conclusions are also consistent with previous literature reports[21,22] that show VO_2 (001) films are coherently strained to TiO_2 (001) substrates up to a critical thickness of 16 nm. We note that all VO_2 films in this work are therefore restricted to thicknesses of <10 nm to avoid any strain-related shits in T_{MIT}.

Supplementary References

1. Yang, Z., Ko, C., Balakrishnan, V., Gopalakrishnan, G. & Ramanathan, S. Dielectric and carrier transport properties of vanadium dioxide thin films across the phase transition utilizing gated capacitor devices. *Phys. Rev. B* **82**, 205101 (2010).
2. Paik, H. *et al.* Transport properties of ultra-thin VO_2 films on (001) TiO_2 grown by reactive molecular-beam epitaxy. *Appl. Phys. Lett.* **107**, 163101 (2015).
3. Rosevear, W. H. & Paul, W. Hall Effect in VO_2 near the Semiconductor-to-Metal Transition. *Phys. Rev. B* **7**, 2109–2111 (1973).
4. Hariki, A., Uozumi, T. & Kuneš, J. LDA+DMFT approach to core-level spectroscopy: Application to 3d transition metal compounds. *Phys. Rev. B* **96**, 045111 (2017).
5. Ghiasi, M. *et al.* Charge-transfer effect in hard x-ray 1s and 2p photoemission spectra: LDA+DMFT and cluster-model analysis. *Phys. Rev. B* **100**, 075146 (2019).
6. Blaha, P., Schwarz, K., Madsen, G. K., Kvasnicka, D. & Luitz, J. wien2k. *An augmented plane wave+ local orbitals program for calculating crystal properties* **60**, 1–302 (2001).
7. Kuneš, J. *et al.* Wien2wannier: From linearized augmented plane waves to maximally localized Wannier functions. *Computer Physics Communications* **181**, 1888–1895 (2010).

8. Mostofi, A. A. *et al.* An updated version of wannier90: A tool for obtaining maximally-localised Wannier functions. *Computer Physics Communications* **185**, 2309–2310 (2014).

9. Brito, W. H., Aguiar, M. C. O., Haule, K. & Kotliar, G. Metal-Insulator Transition in VO_2: A DFT+DMFT Perspective. *Phys. Rev. Lett.* **117**, 056402 (2016).

10. Haverkort, M. W. *et al.* Orbital-Assisted Metal-Insulator Transition in VO_2. *Phys. Rev. Lett.* **95**, 196404 (2005).

11. Hariki, A. *et al.* Satellites in the Ti 1s core level spectra of $SrTiO_3$ and TiO_2. *Phys. Rev. B* **106**, 205138 (2022).

12. Higashi, K., Winder, M., Kuneš, J. & Hariki, A. Core-Level X-Ray Spectroscopy of Infinite-Layer Nickelate: LDA+DMFT Study. *Phys. Rev. X* **11**, 041009 (2021).

13. Biermann, S., Poteryaev, A., Lichtenstein, A. I. & Georges, A. Dynamical Singlets and Correlation-Assisted Peierls Transition in VO_2. *Phys. Rev. Lett.* **94**, 026404 (2005).

14. Okada, K., Uoaumi, T. & Kotani, A. Split-Off State Formation in the Final State of Photoemission in Ti Compounds. *Journal of the Physical Society of Japan* **63**, 3176–3184 (1994).

15. Martens, K., Aetukuri, N., Jeong, J., Samant, M. G. & Parkin, S. S. P. Improved metal-insulator-transition characteristics of ultrathin VO_2 epitaxial films by optimized surface preparation of rutile TiO_2 substrates. *Appl. Phys. Lett.* **104**, 081918 (2014).

16. Mondal, D. *et al.* Atomically-smooth single-crystalline VO_2 (101) thin films with sharp metal-insulator transition. *Journal of Applied Physics* **126**, 215302 (2019).

17. Taguchi, M., Takata, Y. & Chainani, A. Hard X-ray photoelectron spectroscopy: A few recent applications. *Journal of Electron Spectroscopy and Related Phenomena* **190**, 242–248 (2013).

18. Quackenbush, N. F. *et al.* X-Ray Spectroscopy of Ultra-Thin Oxide/Oxide Heteroepitaxial Films: A Case Study of Single-Nanometer VO_2/TiO_2. *Materials* **8**, 5452–5466 (2015).

19. Quackenbush, N. F. *et al.* Nature of the Metal Insulator Transition in Ultrathin Epitaxial Vanadium Dioxide. *Nano Lett.* **13**, 4857–4861 (2013).

20. Eguchi, R. *et al.* Electronic structure of $3d^1$ configuration vanadium oxides studied by soft X-ray and hard X-ray photoemission spectroscopy. *Journal of Electron Spectroscopy and Related Phenomena* **156–158**, 421–425 (2007).

21. Nagashima, K., Yanagida, T., Tanaka, H. & Kawai, T. Stress relaxation effect on transport properties of strained vanadium dioxide epitaxial thin films. *Phys. Rev. B* **74**, 172106 (2006).

22. Fan, L. L. *et al.* Strain Dynamics of Ultrathin VO_2 Film Grown on TiO_2 (001) and the Associated Phase Transition Modulation. *Nano Lett.* **14**, 4036–4043 (2014).
